# Social determinants of adherence to antiretroviral therapy among adolescents and young people living with HIV: a scoping review

**DOI:** 10.1590/1980-220X-REEUSP-2025-0026en

**Published:** 2025-08-18

**Authors:** Camila Moraes Garollo Piran, Mariana Martire Mori, Alana Vitória Escritori Cargnin, Beatriz Sousa da Fonseca, Bianca Machado Cruz Shibukawa, Maria de Fátima Garcia Lopes Merino, João Manuel Graça Frade, Marcela Demitto Furtado

**Affiliations:** 1 Universidade Estadual de Maringá, Programa de Pós-Graduação em Enfermagem, Maringá, PR, Brazil.; 2 Politécnico de Leiria, Escola Superior de Saúde, Leiria, Portugal.; 3 Universidade Federal de Mato Grosso do Sul, Departamento de Enfermagem, Três Lagoas, MS, Brazil.

**Keywords:** HIV, Adolescent, Young Adult, Medication Adherence, Social Determinants of Health

## Abstract

**Objective:**

To map scientific evidence related to social determinants of adherence to antiretroviral therapy among adolescents and young people living with HIV.

**Method:**

Scoping review according to JBI methodology, carried out on 20 databases. The inclusion criteria were: adolescents and young people living with HIV (10 and 24 years old), studies related to adherence to antiretroviral therapy (self-reported adherence, viral suppression, pill counts, or pharmacy refill records).

**Results:**

Thirty-nine studies were identified, published between 1999 and 2024, which allowed mapping the determinants that promote and limit adherence to treatment among adolescents and young people living with HIV, being focused on individual characteristics, lifestyle, social and community networks, living and working conditions, socioeconomic, cultural and environmental conditions (Dimensions 1 to 5).

**Conclusion:**

The social determinants of adherence interact in a complex way, which affects the context of antiretroviral therapy, both positively and negatively, and are directly interconnected with the living conditions of adolescents and young people living with HIV.

## INTRODUCTION

Across the world, adolescents and young people have represented a growing proportion of people living with HIV. In 2022, there were 480,000 adolescents and young people between 10 and 24 years old newly infected with HIV, of which 140,000 were adolescents between 10 and 19 years old. Furthermore, 25% of adolescent girls and 17% of adolescent boys aged 15–19 are from Eastern and Southern Africa, regions most affected by HIV. They were tested in the past 12 months and received their last test result. If current trends continue, there will be 183,000 new HIV infections among adolescents and young adults each year by 2030^(1)^.

Treatment coverage among adolescents and young people living with HIV aged 15 to 24 years was estimated at 55%, significantly lower than the 75% coverage among those who are older^(2)^. Therefore, the need for adherence to HIV treatment is highlighted, as this can save lives when started early and used appropriately. However, it is known that every year several adolescents and young people succumb to AIDS-related complications as a result of poor adherence to or treatment abandonment^(3)^.

Adolescents and young people living with HIV face the burden of the unique physical, psychological and social challenges of adolescence and young adulthood, while also dealing with a stigmatized infectious disease that requires lifelong care, and often do not seek health care assistance^(4)^. This therefore reflects in negative health outcomes among adolescents and young people when compared to other age groups^(5)^.

Thus, it becomes required to adapt services for adolescents and young people in equitable, accessible, acceptable, appropriate, and effective ways^(6)^. In line with this, part of the United Nations Sustainable Development Goals include ending the AIDS epidemic and the goal of achieving universal coverage of sexual and reproductive health services by 2030^(7)^. To achieve these goals, it is necessary to identify the factors that facilitate and limit adherence to HIV treatment among adolescents and young people, helping professionals to develop an individualized and comprehensive care plan for each patient^(2)^.

That said, new initiatives are needed to fight persistent barriers to accessing treatment services among adolescents and young people living with HIV, and strategies have to be created to achieve global goals. Although there is literature on adherence to treatment among this population, it does not show the social determinants of adherence that promote and limit the continuity of treatment, as well as access to health services, which constituted a knowledge gap to be explored. Within this framework, the objective of this study was to map scientific evidence related to social determinants of adherence to antiretroviral therapy among adolescents and young people living with HIV.

## METHOD

### Design of Study

This is a scoping review, according to JBI methodology, which aims to map the current literature, as well as list the main concepts in the area and knowledge gaps, allowing new studies^(8)^. This scoping review was reported as per the checklist *Preferred Reporting Items for Systematic Reviews and MetaAnalyses extension for Scoping Reviews* (PRISMA-ScR)^(9)^. The protocol is registered in the *Open Science Framework* with the identifier osf.io/j8bwa (https://doi.org/10.17605/OSF.IO/EM5TB).

### Guiding Question

To develop the guiding question, the acronym PCC (Population, Concept, and Context) was considered, in which Population (P) is adolescents and young people; Concept (C) is adherence to antiretroviral therapy (ART); and Context (C) is living with HIV/AIDS. Thus, the question was obtained: What is the scientific evidence related to social determinants of adherence to antiretroviral therapy among adolescents and young people living with HIV?

### Inclusion Criteria

Studies in which the population consisted of adolescents and young people, considering the age range between 10 and 24 years, were included^(10)^. Regarding the concept, the studies were related to adherence to antiretroviral therapy, and adherence was explicitly measured using any method, such as subjective measurement (self-reported adherence), physiological methods (viral suppression), or pharmacological measurements (pill counts, pharmacy refill records), with the aim of finding the largest number of studies to identify the knowledge gap. Regarding the context, it was broad, since it considered living with HIV in all aspects, whether biological, psychological, social, cultural or spiritual, and without restriction of location. Furthermore, studies published in full were considered, without restrictions on language, time limit, and methodological design. Articles published in journals and grey literature were also eligible.

### Search Strategy

From the preliminary search in the *Medical Literature and Retrieval System online* (MEDLINE) via *National Center for Biotechnology Information* (NCBI/PubMed) and *Web of Science,* the words in the text contained in the titles and abstracts of the relevant articles were considered to develop the complete search strategy. Subsequently, a pilot of the final search was conducted in two databases, MEDLINE via NCBI/PubMed and EMBASE. After these steps, the definitive search was carried out and, for each database and/or information source included, the search strategy, including the identified index terms and keywords, were adapted. Searches were carried out in the following databases: *Web of Science* (WOS), MEDLINE via NCBI/PubMed, *Science Direct,* Scopus, EMBASE via *Elsevier*, *Scientific Electronic Library Online* (SciELO), COCHRANE and the Latin American and Caribbean Literature in Health Sciences (LILACS); Spanish Bibliographic Index in Health Sciences (IBECS); Nursing Database (BDENF); Western Pacific Region Index Medicus (WPRIM); Peruvian Literature in Health Sciences (LIPECS); *World Health Organization’s Institutional Repository for Information Sharing* (WHO IRIS); Brazilian Bibliography of Dentistry (BBO) via the Virtual Health Library (VHL), accessed through the Journals Portal of the Coordination for the Improvement of Higher Education Personnel (CAPES). Additional strategies considered were the search in gray literature sources: Cybertesis, PeerJ Prepint, MedRxiv, OpenGrey, bioRxiv Preprints, Catalog of Theses and Dissertations, and cross-reference search. The searches were conducted between July and September 2024.

To construct the search strategy, the Boolean operators AND and OR were used, associated with the MeSH (*Medical Subject Headings*), DeCS (Health Sciences Descriptors), Emtree and uncontrolled descriptors (Chart 1).

**Chart 1:** Construction, descriptors/keywords and Boolean operators used in the database – Maringá, PR, Brazil, 2024.

Database	Search strategy
*Web of Science*	ALL = ((((((“Adolescent”) OR (“Young Adult”)) AND ((“adherence to antiretroviral therapy”) OR (“Medication Adherence”) OR (“Cooperation and Adherence to Treatment”) OR (“Patient Cooperation”) OR (“antiretroviral therapy highly active”)) AND ((“HIV Infections”) OR (“HIV”) OR (“Acquired Immunodeficiency Syndrome”))))))
MEDLINE/PubMed	(((“Adolescent” OR “Young Adult”) AND (“adherence to antiretroviral therapy” OR “Medication Adherence” OR “Cooperation and Adherence to Treatment” OR “Patient Cooperation” OR “antiretroviral therapy highly active”) AND (“HIV Infections” OR “HIV” OR “Acquired Immunodeficiency Syndrome”)
*Science Direct*	(“Adolescent” OR “Young Adult”) AND (“adherence to antiretroviral therapy” OR “Medication Adherence” OR “Cooperation and Adherence to Treatment” OR “Patient Cooperation” OR “antiretroviral therapy highly active”) AND (“HIV” OR “Acquired Immunodeficiency Syndrome”)
*Scopus*	((“Adolescent”) OR (“Young Adult”) AND (“adherence to antiretroviral therapy”) OR (“Medication Adherence”) OR (“Cooperation and Adherence to Treatment”) OR (“Patient Cooperation”) OR (“antiretroviral therapy highly active”) AND (“HIV Infections”) OR (“HIV”) OR (“Acquired Immunodeficiency Syndrome”))
*Embase*	(“Adolescent” OR “Young Adult”) AND (“adherence to antiretroviral therapy” OR “Medication Adherence” OR “Cooperation and Adherence to Treatment” OR “Patient Cooperation” OR “antiretroviral therapy highly active”) AND (“HIV Infections” OR “HIV” OR “Acquired Immunodeficiency Syndrome”)
*Scielo*	((((((“Adolescent”) OR (“Young Adult”)) AND ((“adherence to antiretroviral therapy”) OR (“Medication Adherence”) OR (“Cooperation and Adherence to Treatment”) OR (“Patient Cooperation”) OR (“antiretroviral therapy highly active”)) AND ((“HIV Infections”) OR (“HIV”) OR (“Acquired Immunodeficiency Syndrome”))))))
COCHRANE	(“Adolescent” OR “Young Adult”) AND (“adherence to antiretroviral therapy” OR “Medication Adherence” OR “Cooperation and Adherence to Treatment” OR “Patient Cooperation” OR “antiretroviral therapy highly active”) AND (“HIV Infections” OR “HIV” OR “Acquired Immunodeficiency Syndrome”)
LILACS; IBECS; BDENF; WPRIM; LIPECS; WHO IRIS; BBO via BVS	(((((((“Adolescent”) OR (“Young Adult”)) AND ((“adherence to antiretroviral therapy”) OR (“Medication Adherence”) OR (“Cooperation and Adherence to Treatment”) OR (“Patient Cooperation”) OR (“antiretroviral therapy highly active”)) AND ((“HIV Infections”) OR (“HIV”) OR (“Acquired Immunodeficiency Syndrome”))))))) AND (db:(“LILACS” OR “IBECS” OR “BDENF” OR “WPRIM” OR “LIPECS” OR “WHOLIS” OR “BBO”))
*Cybertesis*	(“Adolescent” OR “Young Adult”) AND (“adherence to antiretroviral therapy” OR “Medication Adherence” OR “Cooperation and Adherence to Treatment” OR “Patient Cooperation” OR “antiretroviral therapy highly active”) AND (“HIV Infections” OR “HIV” OR “Acquired Immunodeficiency Syndrome”)
*PeerJ Prepint*	(“Adolescent” OR “Young Adult”) AND (“adherence to antiretroviral therapy” OR “Medication Adherence” OR “Cooperation and Adherence to Treatment” OR “Patient Cooperation” OR “antiretroviral therapy highly active”) AND (“HIV Infections” OR “HIV” OR “Acquired Immunodeficiency Syndrome”)
*MedRxiv*	(“Adolescent” OR “Young Adult”) AND (“adherence to antiretroviral therapy”) AND (“HIV” OR “Acquired Immunodeficiency Syndrome”)
*OpenGrey*	(“Adolescent” OR “Young Adult”) AND (“adherence to antiretroviral therapy” OR “Medication Adherence” OR “Cooperation and Adherence to Treatment” OR “Patient Cooperation” OR “antiretroviral therapy highly active”) AND (“HIV Infections” OR “HIV” OR “Acquired Immunodeficiency Syndrome”)
*Preprints bioRxiv*	(“Adolescent” OR “Young Adult”) AND (“adherence to antiretroviral therapy”) AND (“HIV” OR “Acquired Immunodeficiency Syndrome”)
Catálogo de teses e dissertações	(“Adolescent” OR “Young Adult”) AND (“adherence to antiretroviral therapy”) AND (“HIV” OR “Acquired Immunodeficiency Syndrome”)

### Source Selection

After searching the information sources, the results were exported to the application *Rayyan*® of *Qatar Computing Research Institute* (QCRI). Thus, of the 10,540 publications, 1,760 were excluded due to duplication. Subsequently, 8,779 studies were analyzed, being selected by title and abstract by two independent reviewers to maintain the blinding process; subsequently, the selected studies were compared and a consensus was reached. There was no need for a third reviewer. After that, 8,708 were excluded because they did not meet the inclusion criteria.

During the full reading, an in-depth reading was carried out applying the eligibility criteria. The study information was organized and stored in electronic spreadsheets to ensure better interpretation and comparison between reviewers.

The articles included in the final sample were organized into an instrument in *Microsoft Excel*®, which is adapted in accordance with the JBI methodology^(8)^ and contained the data characterizing the publications (authors, year, title, language, country, source of information, method, adherence measure, population, main results).

### Data Analysis, Extraction and Presentation

**Figure 1:**
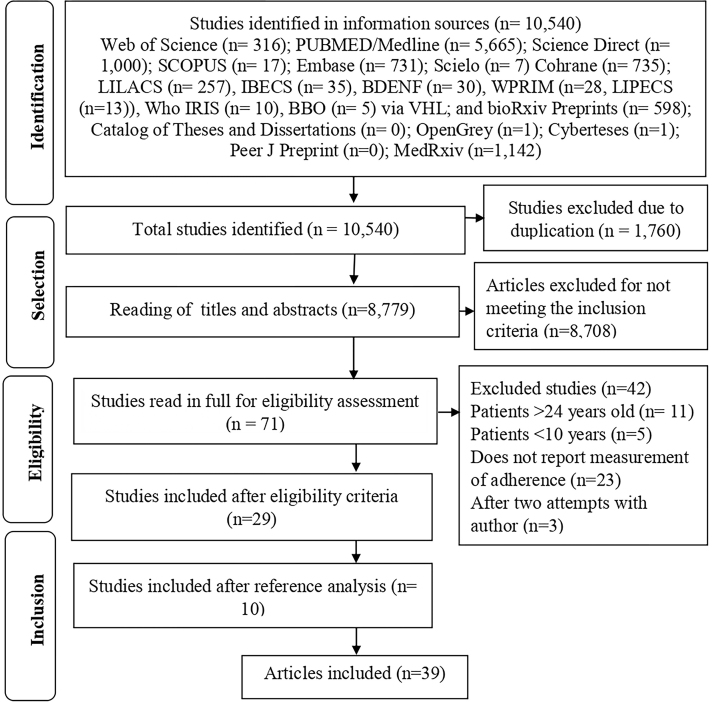
Flowchart for identifying and selecting articles included in the scoping review. Maringa, PR, Brazil, 2024.

Source: adaptation PRISMA-ScR Checklist^(9)^.

A careful reading was carried out to classify the texts and, subsequently, the results were extracted. In addition, a manual search was performed to verify the list of references of the studies included in the review, which resulted in the inclusion of ten more articles. This stage was intended to find studies that were not linked to databases. At the end, 39 publications were part of this study. Figure 1 shows the process of searching, excluding, and selecting publications according to PRISMA^(9)^.

All information from the included studies was stored in spreadsheets and analytical charts. The included studies were analyzed in light of the Social Determinants of Health^(11)^, which facilitated the process of interpreting and comparing the productions, consequently helping in the description of evidence and data mapping, which were related to the description of the social determinants of adherence among adolescents and young people living with HIV. The determinants were presented in a chart.

Considering that this study is a scoping review that used already available publications, assessment by a Research Ethics Committee is not required. However, the research was developed considering the ethical aspects related to the selected articles authorship.

## RESULTS

The application of search strategies in information sources allowed the identification of 10,540 publications. During the title and abstract selection, 71 studies were obtained for full analysis, and of these, after applying the eligibility criteria, 39 studies were selected, whose results answered the guiding question^(12–50)^.

### General Characteristics of The Included Studies

It was observed that 37 (94.87%)^(12–21,23–50)^ presented the text available in English, one (2.56%) in French^(22)^ and one (2.56%) in Portuguese^(18)^. The publication period covered the years 1999 to 2024, with 20 publications relating to the period 2017–2024^(31–50)^. Of the 39 studies, 17 were reported to have been developed on the African continent: five in Uganda^(24,30,39,45–46)^, three in South Africa^(42,44,50)^, two in Botswana^(21,37)^, Kenya^(36,40)^, Ethiopia^(47,48)^ and Zambia^(35,36)^, one in each of the following: Zimbabwe^(27)^, Sub-Saharan Africa^(28)^, Malawi^(32)^, Cameroon^(41)^, Nigeria^(43)^ and Eswatini^(49)^; in North America, 15 studies were developed, all from the United States of America^(12–17,19–20,23,25,26,29,31,33,38)^; in Latin America, only one study was carried out in Brazil^(18)^; and in Europe a publication from Belgium was found^(22)^.

### Objectives, Methods, Adherence Measures and Participants of Included Studies

Regarding the objectives of the studies, most publications were designed to determine the factors associated with adherence to HIV treatment among adolescents and young people, in relation to the promoting and limiting outcomes of the continuity of the follow-up. Other publications have focused on assessing adherence, as well as patients’ experiences regarding barriers and facilitators to adherence.

Regarding the methods used in the studies, it was noted that 28 (71.79%) are quantitative^(12–17,19–23,26–30,32,34,38–41,44,46,47)^, 23 with cross-sectional design^(12–16,20–23,26–30,32,34,38–42,44,46,47,49,50)^, three prospective cohorts^(17,19,50)^, one retrospective^(42)^, and one, a retrospective longitudinal^(49)^ cohort; five (12.82%) were qualitative^(18,24,31,37,45)^, one phenomenological^(45)^, and four were descriptive^(18, 24, 31, 37)^; three (7.69%) mixed methods^(35,36,43)^, with only one categorized as convergent parallel^(24)^, two (5.12%) systematic reviews with meta-analysis^(25, 48)^, and one (2.58%) of quantitative and qualitative approach^(33)^.

Regarding adherence measures, 51 forms were mentioned, 35 (68.62%) of which were subjective medication measures aimed at self-reported adherence^(12,14–17,19–46,48,50)^, nine (17.64%) of physiological methods^(14,18,22,23,25,33,42,49,50)^, and seven (13.72%) of pharmacological measurements^(13,21,24,25,45,48,50)^. Of the 39 studies, it was observed that 28 (71.80%) used only one measure of adherence^(12–17,19–21,26,27,29–32,34–40,43–47,50)^, eight (20.51%) used two medications^(18,22–24,41,42,48,49)^, and three (7.69%) studies used three adherence measures^(25,28,33)^. Regarding the participants, there was only one study carried out with adolescents and young people together with their families^(33)^. With respect to the participants’ age range, it was noted that 15 (38.47%) studies included adolescents and young people (10–24 years old)^(12,13,16,18–20,22,23,26,29,31,38,43,45,47)^ and 24 (61.53%) only adolescents (10–19 years old)^(14,15,17,21,24,25,27,28,30–37,39–42,44,46,48–50)^.

### Conceptual Framework

The promoting and limiting factors identified in the included studies were organized into five dimensions related to the determinants influencing adherence to treatment among adolescents and young people living with HIV: Dimension 1 – Individual Characteristics; Dimension 2 – Lifestyle and individual behaviors; Dimension 3 – Social and community networks; Dimension 4 – Living and health conditions; Dimension 5 – Socioeconomic, cultural, and environmental conditions. The determinants were summarized in Chart 2.

**Chart 2:** Summary of determinants identified in the studies included (n = 39) in the scoping review. Maringa, PR, Brazil, 2024.

Dimension of the determinants	Social determinants of adherence to antiretroviral therapy among adolescents and young people living with HIV	
	Promoters	Limiting factors
Dimension 1: Individual Characteristics	**1. Understanding health and treatment:** Increased health literacy^(23)^ Knowledge of serological status^(25,48)^ Understanding the disease^(26)^ Understanding the relevance of medication^(31)^ Knowledge of the effectiveness of ART^(36)^ Perceived usefulness of HIV medications^(45)^ **2. Motivation and self-efficacy:** They hope to extend their life by at least 10 years^(12)^ Personal goal-oriented motivators^(31)^ I wish to be healthy and live^(37)^ Motivational readiness for ART^(38)^ Greater self-efficacy^(38, 40)^ Ways to self-motivate to achieve adherence^(43)^ **3. Treatment management strategies:** Use of memory aids^(31)^ Use of reminders^(43)^ Concealment strategies^(43)^ They instinctively know when it’s time to use their antiretrovirals^(43)^ **4. Personal attitudes and beliefs:** Fear of negative consequences of non-adherence^(34)^ Life satisfaction^(46)^	**1. Age- and development-related factors:** Young people with more advanced age^(17,33,42,46)^ Stunted growth^(25)^ **2. Forgetfulness and lack of consistency:** Forgetfulness^(20,22,32–34,37)^ Teenager never takes ART while away^(25)^ Having lost doses of ART due to failure to collect them at the pharmacy^(21)^ **3. Lack of motivation and refusal of ART:** Not feeling like taking medication^(20)^ Not wanting to be reminded of HIV infection^(20)^ Low self-efficacy in treatment^(32)^ Teenager does not worry about ART^(25)^ **4. Contextual factors:** Male sex^(21,25,46)^ Female Sex^(25)^ Being a double orphan^(25)^ Lack of basic knowledge about HIV^(35)^ Adolescents with horizontally acquired HIV^(42)^
Dimension 2: Lifestyle and individual behaviors	**1. Stability and routine of life:** Stability of living situations^(13)^ Lifestyle and existence of a daily routine^(31)^ Adolescents on low-start and increasing adherence trajectories^(50)^ **2. Mental health and well-being:** Well-being and low psychological distress^(29,38,44)^ Less alcohol use^(17,29)^	**1. Mental health issues:** Depression^(14,16,17,19,24,35)^ Psychological suffering^(20,29)^ Low mental health score^(25)^ Unitary increase in psychological distress^(28)^ Emotional problems^(31)^ Feeling depressed/overwhelmed^(32)^ Self-reported unhappiness^(33)^ **2. Substance Use:** Substance use^(12,20,38)^ Marijuana use^(16,29)^ Alcohol use^(32)^ **3. Distracting behaviors and activities:** Being busy with other things^(32)^ Having a boyfriend/girlfriend, frequent online chatting^(33)^ **4. Non-adherence behaviors:** Intentional refusal to take medication^(43)^ Being sexually active^(25)^
Dimension 3: Social and community networks	**1. Family Support:** Family support^(18)^ Administration of medication by parents^(25)^ Family cohesion^(39)^ Adolescent-caregiver communication^(39)^ They disclosed their HIV status(mother)^(12)^ Having a caregiver with a partner^(25)^ Have the person in charge present at each clinical meeting^(27)^ **2. Support by the health professional:** Support from the healthcare team^(18)^ Healthcare professionals providing support^(24)^ Adolescents who were taught by a health professional how to take ART^(25)^ Comfort in asking questions to the healthcare professional^(27)^ **3. Peer and group social support:** Peer support^(24)^ Advice from healthcare professionals^(24)^ Social and emotional support and peer group counseling^(33)^ Support from treatment partners or family^(43)^ Social support and counseling services^(36)^ Community Resources for HIV^(36)^ Greater social support^(38,45)^ **4. Coping Strategies and Resources:** Coping (proactive coping strategies; turning to family; spiritual coping; professional help)^(19)^ Support in the home environment^(37)^ Not taking medicine at school^(17)^ Teenager is unaware of caregiver’s health problems^(25)^	**1. Caregiver characteristics:** Caregiver being widowed^(25)^ Caregiver not having a religious practice^(25)^ Low caregiver involvement^(25)^ Low level of caregiver education^(25)^ Low intellectual capacity assessed by the caregiver^(33)^ Having grandparents or relatives as the primary caregiver^(33)^ Having a grandparent as the primary caregiver^(46)^ Caregiver being the only one who knows the child’s HIV status^(25)^ **2. Impaired family and relational dynamics:** Having a disorganized family and in loco parentis arrangements^(25)^ Tense relationships with caregivers^(33)^ Lack of care, support and love from the father^(43)^ Blame whoever contaminated the mother^(43)^ **3. Violence and trauma:** Witnessing or experiencing violence at home^(32)^ Loss of mother^(35)^ **4. Communication and Collaboration:** Poor communication with the doctor^(33)^ Lack of collaboration between organizations and social norms^(36)^
Dimension 4: Living and health conditions	**1. Clinical aspects of treatment:** CD4 level ≥ 500 cells/mm^3(13)^ Increased CD4+ counts^(34)^ Viral loads <1000 copies/ml^(40)^ Undetectable baseline viral load^(49)^ Taking cotrimoxazole prophylaxis in conjunction with ART^(25)^ Number of medications prescribed^(13)^ Absence of side effects and nutrition/diet^(31)^ Privacy to take the medicine^(31)^ Characteristics of the medicines (number and size of tablets, regimen)^(31)^ Once a day dosage^(40)^ **2. Resources for living conditions and health:** Participate in group sessions led by a professional facilitator^(27)^ Food supply^(24)^ Enough food to eat while taking the medication^(44)^ Adequate supply of medicines^(36)^ Those who received care for their concern within the unit^(47)^ **3. Accessibility to health services:** Short waiting time^(24)^ Living closer to a clinic^(30)^ Availability/accessibility (having transportation)^(24,31)^ Enough money to travel to the clinic^(44)^ Travel close to home^(32)^ Availability of free services^(45)^	**1. Poor housing and food conditions:** Homelessness or lack of housing^(12,13)^ Lack of adequate nutrition^(36)^ **2. Aspects related to treatment:** Many pills^(12,24)^ Take 3 or more tablets per day^(25)^ Difficulty in administering medication by the caregiver^(25)^ Medication administered by the adolescent^(25)^ Adolescents in the most advanced stage of HIV, presenting AIDS^(17)^ Previous history of opportunistic infection^(47)^ RNA viral load ≥1000 copies/ml^(33)^ Detectable baseline viral load^(49)^ Not following the prescribed regimen^(28)^ **3. Side effects and treatment burden:** Medication-related adverse effects^(15,24,35,41,43)^ Medication intolerance^(25)^ Drug fatigue^(24,31)^ Tired of taking medication every day for the rest of their lives^(43)^ Perceived burden of treatment^(45)^ Perceived effect of ART on physical appearance^(43)^ **4. Low access and support from health services:** Living far from work, whether in an urban or rural area^(25)^ Living in remote areas^(50)^ Low satisfaction with health care^(19)^ Lack of specific services for adolescents^(36)^ Caregiver does not pay a fee for treatment^(25)^ Complications in everyday routines^(15)^ Missing a doctor’s appointment in the last 6 months^(32)^ Missing one or more doses of ART in the last week^(36)^ Long waiting time at the clinic^(34)^
Dimension 5: Socioeconomic, cultural and environmental conditions	**1. Economic resources:** Larger asset ownership, specifically family ownership of seven or more tangible assets^(30)^ Greater economic advantage in household goods ownership, financial savings, and caregiver employment^(30)^ Improvements in economic well-being ^(44)^ within and between people **2. Stigma Reduction:** Reducing internalized HIV stigma^(44)^	**1. Stigma and discrimination:** Prejudice, discrimination and stigma^(18,24,34,36,37)^ Internalized stigma^(41)^ Feeling stigmatized by people outside and inside the home^(32)^ Perceived resulting stigma and discrimination^(45)^ **2. Fears related to disclosure:** Fear of being seen taking antiretroviral medication^(22,24)^ Fear of disclosure^(31,36,37,43)^ Fear of disclosing HIV status to others, especially boys/girlfriends^(33)^ **3. Socioeconomic conditions:** Poverty^(24,36)^

Source: Research data, 2024.

**Figure 2:**
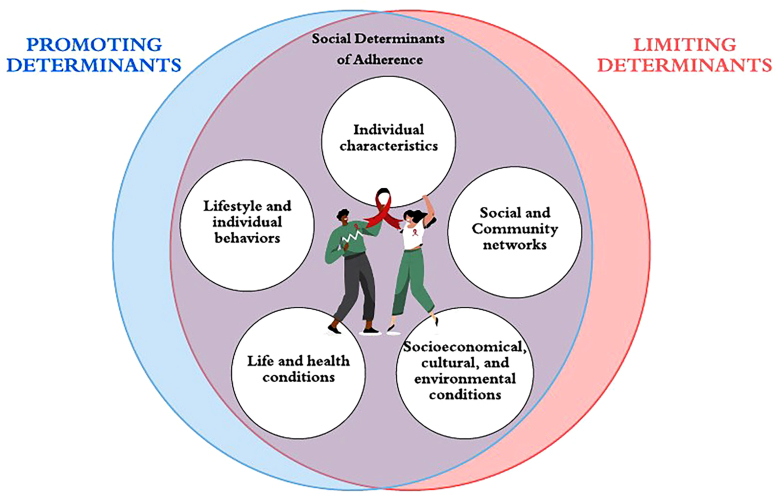
Conceptual synthesis of the results from the final sample of social determinants of adherence among adolescents and young people living with HIV. Maringá, Paraná, Brazil, 2024.

Source: Research data, 2024.

This review found that factors related to individual characteristics, lifestyle, social and community networks, living and working conditions, socioeconomic, cultural and environmental conditions of adolescents and young people influenced adherence to HIV treatment, being constantly shaped by being multilevel, that is, interacting in a complex way. Thus, the social determinants of adherence affect the context of antiretroviral treatment, both positively and negatively, and are directly linked to the living conditions of adolescents and young people living with HIV (Figure 2).

## DISCUSSION

The studies included in the review cover a wide range of countries and continents, such as North America, Latin America, Africa, Asia and Europe, and are high, middle and low average income per capita countries. Geographical and cultural diversities are important to understand how different social, economic, and cultural contexts influence adherence to antiretroviral therapy among adolescents and young people. Adapting intervention strategies must consider these contextual variations^(51)^.

Regarding the temporality of the materials found, a significant part of the studies were published between 2017 and 2024. This fact is especially due to the launch of the United Nations General Assembly Political Declaration on ending AIDS and reducing new HIV infections. Since the launch of this declaration in 2016, countries around the world have committed to accelerating their actions to combat HIV and AIDS to end the epidemic by 2030^(52)^.

It is clear that the minority of studies used a qualitative approach, and are essential for exploring the barriers and facilitators of adherence to ART among adolescents and young people. The qualitative approach allows for a more in-depth analysis of the perceptions, experiences and social contexts of adolescents and young people living with HIV, in addition to elucidating psychosocial and cultural issues that affect adherence, which may not be captured by quantitative studies^(53,54)^.

Regarding the dimension of the individual characteristics of adolescents and young people living with HIV, it was noted that greater knowledge of serological status and of the importance of antiretroviral therapy are determinants that promote adherence. Therefore, if young people have access to information and understand it, it means they have knowledge about HIV/AIDS and ART. This evidence helps managers make decisions about training and keeping counselors and health professionals up to date so that they can, in turn, communicate accurate information to adolescents and young people^(55)^.

Thus, health literacy has been a factor that influences adherence to treatment for numerous infections and diseases, especially HIV and AIDS. Patients who receive information appropriate to their level of understanding have significant results throughout treatment^(56,57)^. The HIV knowledge and beliefs of adolescents and young people living with HIV play an essential role in their experience^(58)^.

Linked to these individual factors, personal goals and the desire to live, combined with satisfaction with life, are aspects that promote adherence to treatment. A cross-sectional study carried out in China showed that people who accept their HIV diagnosis adhere to treatment and carry it out correctly. Consequently, they have a higher quality of life and life expectancy^(59)^. Acceptance of HIV as a chronic condition helps to change the behavior of adolescents and young people and, in turn, leads them to change their lifestyle, with the aim of becoming undetectable and non-communicable (U = NC)^(58)^.

However, some personal characteristics hinder the implementation of correct treatment, such as early adults and being male. Thus, being young is already a factor, since their responsibility is seen as a barrier, related to the fatigue of undergoing continuous treatment, in addition to comparison with people from the same social circle and involvement in other activities and habits specific to the age group. Furthermore, young men have more difficulty seeking help from health services, due to their own gender stereotype, undermining men’s engagement across the care cascade. Therefore, as this is a phase in which they receive less social and family support, older adolescents and young people are more likely to not adhere to treatment or have poor adherence^(57)^.

Furthermore, a systematic review and meta-analysis conducted with orphaned adolescents showed that orphanhood status is a significant barrier to ART adherence, especially in low-income countries. Dependence on caregivers, which may be inconsistent or insufficient, compounds this challenge, indicating the need for interventions that increase social support and improve caregiver education about the importance of treatment adherence^(60)^.

Added to this, we see that many teenagers and young people present occasional lapses in taking their antiretroviral medications due to forgetfulness, which is often caused by the psychological stress of having the infection and/or illness and having to take the medication daily^(61)^.

Regarding the dimension of lifestyle and individual behaviors, it was noted that psychological well-being is an important determinant that must be taken into account in people living with HIV, especially adolescents and young people, as it is one of the factors that contribute to treatment success. These individuals dedicate themselves to treatment with the aim of prolonging their lives and adopting a healthy lifestyle, with a routine that minimizes psychological suffering, capable of experiencing situations of resilience in the face of the condition^(62)^.

However, mental disorders and psychological distress, such as anxiety and depression, are common in the lives of these young people, which, combined with the abuse of legal and illegal substances, can lead to treatment abandonment^(62)^. Therefore, experiencing emotional dissonance and having difficulty embracing one’s own identity shows that mental health is fragile, impacting treatment^(58)^.

In the social and community networks dimension, it was noted that support, whether social, from the family or from a health professional, is a determinant that contributes positively to adherence to antiretroviral therapy among adolescents and young people living with HIV. Social relationships are necessary for all individuals, especially for people living with a chronic illness, where a support network and trusting relationships are important for coping with the disease^(63)^. A study conducted with Peruvian adolescents highlighted how social and cultural factors, including family support and social dynamics, influence adherence to treatment. The use of social ecological models in these studies emphasizes the need to consider multiple levels of influence, from the individual to the community level, when designing interventions to improve ART adherence^(53)^.

Moreover, trustful relationships established with healthcare professionals are important for some patients, being, in most cases, the only source of support and guidance^(57)^. A systematic review of the literature identified that counseling and education about antiretroviral therapy are facilitators of treatment adherence, when considering the patient in their entirety, including the individual’s ethnic, cultural, socioeconomic aspects and educational level^(56,57)^.

In this same sense, peer support is important for facing treatment; however, some young people feel embarrassed and different when they relate to partners who do not have the same condition, not finding mutual support^(64)^. In this way, some adolescents and young people living with HIV continually seek and may have received support from other significant people and the community in their lives, making treatment and diagnosis something easier in their daily lives^(58)^.

Damaged family dynamics, the relationship between a young person living with HIV and other family members, and trauma in the home environment are still determining factors that have negatively influenced adherence to antiretroviral therapy. Stigma and prejudice, in most cases, come from the family itself, and contribute to depressive symptoms and suicidal ideation^(57)^.

Therefore, it is essential to invest in social support for these patients, together with their family members who know about the diagnosis, and the healthcare team, so that their quality of life and survival can be improved through adherence to ART^(65)^. Furthermore, it is highlighted that only one study^(33)^ brought the perspective of the family caregiver. This result highlights the need to develop new studies in this area with the aim of improving adherence to antiretroviral therapy together with the support of family members of adolescents and young people living with HIV.

It can be seen that in the dimension of living and health conditions, the promoting determinants are related to treatment and accessibility to health services. It is possible to identify that the viral load <1000 copies/ml or undetectable and a CD4+ count level ≥ 500 cells/mm^3^ or increased is associated with adherence, as young people feel encouraged to continue treatment. HIV suppression is an essential strategy for optimizing the health and well-being of those living with the infection. Routine viral load (VL) and CD4+ count testings are essential to improve treatment opportunities and are a particularly useful tool among groups at high risk of virological failure, such as adolescents^(66)^.

A study carried out in the United Kingdom and Ireland showed that in young people who started ART before the age of 10, the CD4+ count decreased from the age of 10 onwards, and that viral suppression <400 copies/ml over time presented mean CD4+ counts that approached mild immunodeficiency (350–500 cells/mm^3^) at the age of 20^(67)^.

Adverse effects of antiretrovirals have been considered a limiting factor in ART adherence, as adolescents and young people living with HIV often struggle with self-management of treatment. Many have symptoms due to HIV disease, comorbid conditions, and/or adverse effects of antiretrovirals^(62)^.

Adverse effects directly affect adherence, as they affect the daily lives of adolescents and/or young people and, consequently, their quality of life. Thus, to be able to neutralize barriers while improving facilitators, treatment optimization with dolutegravir (DTG)-based regimens, approaches oriented by peers, such as youth and adolescent peer supporters, and differentiated service delivery models including multi-month dispensing is an excellent strategy to minimize the impact of treatment on their lives^(68,69,70)^.

Geographical distance has been preponderant in adherence to HIV treatment and care, mainly due to its chronicity. In general, it is a factor that limits access to health services, being a barrier that directly interferes with the rates of adherence and abandonment of treatment when the distances are greater^(65,71)^. The lack of financial resources also affects access to treatment and, combined with distance, can interfere with both the individual’s own health outcomes and the dynamics of services, as they end up missing appointments and routines or abandoning treatment^(65,72)^.

Likewise, accessibility to health services associated with distance from residence and individuals’ socioeconomic factors are barriers to good adherence to ART, increasing the adolescents’ vulnerability^(57)^. Conversely, when adolescents and young people live close to the HIV service, they end up seeking the service more frequently, precisely because they do not need to pay for the service and because they live close to the place where they can consult and collect their medication^(30,45)^.

Therefore, it is important that specialized HIV services be integrated with other levels of health services, especially those that make up primary care, as the lack of integration has hindered the development of prevention and promotion strategies and actions for this population^(71)^.

That said, it is essential that health services focused on HIV also monitor absences and their reasons, and thus seek alternatives for greater adherence to follow-up consultations and the use of ART. Furthermore, patients must be made aware so that they can take responsibility for their own health, so that they can get involved in their care and, consequently, improve their quality of life^(73)^.

The dimension of socioeconomic, cultural, and environmental conditions highlights the need for society and adolescents and young people living with HIV to reduce stigma. A study conducted in the Philippines showed that dealing with stereotypes about HIV contributed to the way adolescents and young people assume the judgments of others (perceived stigma) and thus internalize the stigma that changes their beliefs about themselves and the disease^(58)^. It is also clear that stigma and discrimination are caused by misinformation about the disease and come from both themselves and others around them^(58)^.

There is evidence that prejudice, discrimination, stigma, and fear of disclosing the diagnosis have significantly impaired adherence to antiretroviral therapy. A study carried out with adolescents in Ethiopia highlighted that the barriers of stigma, prejudice, discrimination and fear of disclosing the diagnosis have been the most prevalent. Thus, the research encourages interventions aimed at improving social support and reducing stigma, prejudice and discrimination, thus enabling adolescents and young people to leave fear aside and be able to live better with HIV^(74)^.

Recent qualitative studies, such as those carried out in sub- Saharan African countries and Peru^(53,54)^, highlight the critical role of stigma, fear of disclosure and emotional challenges faced by adolescents, which are determinants for optimal adherence to ART. It is noteworthy that the COVID-19 pandemic has exacerbated these barriers, increasing social isolation and hindering access to health services, which has had a negative impact on adherence to ART in several regions of the world^(75)^.

Poverty has also been a deeply stigmatized phenomenon due to aspects of social inequality, in addition to being a factor that contributes to food and housing insecurity, negatively impacting adherence to treatment. Therefore, it becomes essential to address resource insecurity to get to the heart of HIV-related stigma^(76)^.

Adolescents and young people living with HIV in low- and middle-income countries come from different geographic and socioeconomic backgrounds; consequently, they are at higher risk of psychosocial, developmental and comorbid problems, becoming a particularly vulnerable group in terms of treatment adherence. In addition, there are also discrepancies within resource scenarios, lack of structure, and ineffective communication among healthcare providers^(77)^.

All of these dimensions have implications for public health promotion strategies in the context of treatment adherence among adolescents and young people living with HIV, including the design and development of family-based, social and health professional interventions. Effective strategies must be multifaceted, addressing behavioral, social, and structural factors to minimize poor adherence and loss to follow-up.

In this context, international efforts are being made with the aim of ending HIV as a public health problem by 2030, with the proposal that social inequalities be combated and intersectoral actions be expanded. Achieving the 95-95-95 target should be the goal of all countries in the world, and this requires the availability of timely testing and treatment, combined with viral load remission. Along with the adherence to drug therapy, it is also necessary for adolescents and young people living with HIV to adopt an active self-care approach, by undertaking a healthy lifestyle, attending appointments, taking tests, collecting medication on the scheduled date, as well as taking the medication correctly daily. This way, the focus of care is on adolescents and young people living with HIV; therefore, health professionals must encourage an active stance in their own treatment^(78)^.

### Study Limitations

As a limitation, it should be highlighted that all types of adherence measures were considered, which implies possible biases in the sample as well as the methodological heterogeneity associated with each type of measure, making it impossible to generalize the data. Another limitation is the non-inclusion of texts in other indexing databases; therefore, it is recognized that important published research may have been omitted with the search strategy used in this review. Nevertheless, it should be noted that all scientific rigor required for a scoping review was followed in accordance with JBI recommendations. Furthermore, the results of the conceptual synthesis should be interpreted with caution, since they present different epidemiological and socioeconomic scenarios, as well as measures and strategies implemented, operational and organizational aspects, and the format of the health system in each country.

### Advances in Nursing and Health

Health professionals, especially nurses who work in HIV outpatient clinics and Primary Health Care (PHC) serving adolescents and young people, can benefit from the results, since the synthesized conceptual framework brings to light everyday situations that directly influence treatment. Consequently, with promoting and limiting determinants being recognized, the process of searching for strategies makes the reduction of rates of poor adherence and non-adherence to treatment less arduous and more specific. Based on this study, new research can be carried out to eliminate the factors that are known to limit the treatment of adolescents and young people living with HIV on several fronts and in different contexts.

## CONCLUSION

Mapping the determinants that promote and/or limit adherence to antiretroviral therapy among adolescents and young people living with HIV allowed mitigating the (re)structuring of public policies in relation to clinical practice, especially in health services that meet the needs of this public, in reducing social inequalities and promoting adherence to treatment, consequently leading to the achievement of goals by 2030. The summarized findings highlight that adherence to treatment is based on personal beliefs and knowledge, routine dynamics in the family environment, and access to health services. This study also indicates the need for new research that seeks to capture the diversity of factors that reflect on the lived experience and behaviors related to adherence to antiretroviral treatment in the context of adolescence and youth.

## DATA AVAILABILITY

The dataset supporting the findings of this study is not publicly available, however, all data supporting the findings of this study are available upon request to the corresponding author.
