# Supplementary material for: Social determinants of adherence to antiretroviral therapy among adolescents and young people living with HIV: a scoping review
Source: Rev Esc Enferm USP. 2025 Aug 18;59:e20250026. doi: 10.1590/1980-220X-REEUSP-2025-0026en (PMC12372771; doi:10.1590/1980-220X-REEUSP-2025-0026en)
Supplement: Translation

# Translation: Determinantes sociais da adesão à terapia antirretroviral entre adolescentes e jovens vivendo com HIV: *scoping review*

**DOI:** 10.1590/1980-220X-REEUSP-2025-0026pt

**Authors:** Camila Moraes Garollo Piran, Mariana Martire Mori, Alana Vitória Escritori Cargnin, Beatriz Sousa da Fonseca, Bianca Machado Cruz Shibukawa, Maria de Fátima Garcia Lopes Merino, João Manuel Graça Frade, Marcela Demitto Furtado

**Affiliations:** 1 Universidade Estadual de Maringá, Programa de Pós-Graduação em Enfermagem, Maringá, PR, Brasil.; 2 Politécnico de Leiria, Escola Superior de Saúde, Leiria, Portugal.; 3 Universidade Federal de Mato Grosso do Sul, Departamento de Enfermagem, Três Lagoas, MS, Brasil.

**Keywords:** HIV, Adolescente, Adulto Jovem, Adesão à Medicação, Determinantes Sociais da Saúde

## Abstract

**Objetivo:**

Mapear as evidências científicas relacionadas aos determinantes sociais da adesão à terapia antirretroviral entre adolescentes e jovens vivendo com HIV.

**Método:**

*Scoping review* de acordo com metodologia JBI para revisão de escopo, realizada em 20 bases de dados. Os critérios de inclusão foram: adolescentes e jovens vivendo com HIV (10 e 24 anos), estudos relacionados a adesão à terapia antirretroviral (adesão autorrelatada, supressão viral, contagem de comprimidos ou registros de reabastecimento de farmácia).

**Resultados:**

Identificaram-se 39 estudos, publicados entre 1999 e 2024, que possibilitaram mapear os determinantes promotores e limitantes da adesão ao tratamento entre adolescentes e jovens vivendo com HIV, sendo voltados às características individuais, estilo de vida, redes sociais e comunitárias, condições de vida e trabalho, condições socioeconômicas, culturais e ambientais (Dimensões de 1 a 5).

**Conclusão:**

Os determinantes sociais da adesão interagem de forma complexa, o que afeta o contexto da terapia antirretroviral, tanto de forma positiva quando negativa, e estão diretamente interligados às condições de vida dos adolescentes e jovens vivendo com HIV.

## INTRODUÇÃO

No mundo, os adolescentes e jovens têm representado uma parcela crescente de pessoas vivendo com HIV. Em 2022, houve 480.000 adolescentes e jovens entre 10 e 24 anos recém infectados pelo HIV, sendo que 140.000 foram adolescentes entre 10 e 19 anos. Além disso, 25% das meninas adolescentes e 17% dos meninos adolescentes de 15 a 19 anos são da África Oriental e Meridional, regiões mais afetadas pelo HIV, os quais foram testados nos últimos 12 meses e receberam o resultado do último teste. Se essa tendência atual se mantiver, em 2030 haverá 183.000 novas infecções anuais por HIV entre adolescentes e jovens^(1)^.

Estima-se que a cobertura do tratamento entre adolescentes e jovens vivendo com HIV na faixa etária de 15 a 24 anos foi de 55%, significativamente menor do que a cobertura de 75% entre aqueles com mais idade^(2)^. Dessa forma, ressalta-se a necessidade da adesão ao tratamento de HIV, sendo este capaz de salvar vidas quando iniciado precocemente e usado adequadamente. Entretanto, sabe-se que anualmente vários adolescentes e jovens sucumbem a complicações relacionadas à aids em decorrência da má-adesão ou abandono ao tratamento^(3)^.

Os adolescentes e jovens vivendo com HIV deparam-se com o fardo dos desafios físicos, psicológicos e sociais únicos da adolescência e juventude, sendo que ao mesmo tempo lidam com uma doença infecciosa estigmatizada que requer cuidados ao longo da vida, e muitas vezes não buscam assistência no serviço de saúde^(4)^. Dessa forma, isso reflete em resultados de saúde negativos entre adolescentes e jovens quando comparado a outros grupos etários^(5)^.

Assim, torna-se necessário adequar os serviços para os adolescentes e jovens, de formas equitativas, acessíveis, aceitáveis, apropriadas e eficazes^(6)^. Em consonância a isso, parte dos Objetivos de Desenvolvimento Sustentável da Organização das Nações Unidas incluem o fim da epidemia de aids e a meta de atingir a cobertura universal de serviços de saúde sexual e reprodutiva até 2030^(7)^. Para que essas metas sejam alcançadas, é necessário identificar os fatores que facilitam e limitam a adesão ao tratamento de HIV entre adolescentes e jovens, contribuindo com os profissionais para elaborarem um plano de cuidado individualizado e integral para cada paciente^(2)^.

Posto isto, são necessárias novas iniciativas que combatam as barreiras persistentes de acesso aos serviços de tratamento entre os adolescentes e jovens vivendo com HIV, e estratégias sejam criadas para o alcance das metas globais. Embora haja literatura sobre a adesão ao tratamento entre esse público, os mesmos não evidenciam os determinantes sociais da adesão que promovem e limitam a continuidade do tratamento, assim como o acesso ao serviço de saúde, o que se constituiu como uma lacuna de conhecimento a ser explorada. Nesse sentido, o objetivo deste estudo foi mapear as evidências científicas relacionadas aos determinantes sociais da adesão à terapia antirretroviral entre adolescentes e jovens vivendo com HIV.

## MÉTODO

### Tipo de Estudo

Trata-se de uma revisão de escopo de acordo com metodologia JBI para revisão de escopo que tem por finalidade mapear a literatura atual, assim como elencar os principais conceitos da área e lacunas do conhecimento, possibilitando novos estudos^(8)^. Esta revisão de escopo foi reportada conforme o checklist *Preferred Reporting Items for Systematic Reviews and MetaAnalyses extension for Scoping Reviews* (PRISMA-ScR)^(9)^. O protocolo está registrado na *Open Science Framework* com o identificador osf.io/j8bwa (https://doi.org/10.17605/OSF.IO/EM5TB).

### Questão Norteadora

Para elaboração da questão norteadora, considerou-se o acrônimo PCC (População, Conceito e Contexto), em que a População (P) são adolescentes e jovens; Conceito (C) é a adesão a terapia antirretroviral (TARV); e Contexto (C) é viver com HIV/aids. Assim, obteve-se a questão: Quais são as evidências científicas relacionadas aos determinantes sociais da adesão à terapia antirretroviral entre adolescentes e jovens vivendo com HIV?

### Critérios de Inclusão

Foram incluídos estudos em que a população era composta por adolescentes e jovens, considerando a faixa etária entre 10 e 24 anos^(10)^. Quanto ao conceito, os estudos estavam relacionados com a adesão à terapia antirretroviral, e a adesão foi medida explicitamente usando qualquer método, como por exemplo medição subjetiva (adesão autorrelatada), métodos fisiológicos (supressão viral) ou medições farmacológicas (contagem de comprimidos, registros de reabastecimento de farmácia), tendo como finalidade encontrar o maior número de estudos para identificação da lacuna do conhecimento. Já referente ao contexto, foi amplo, uma vez que considerou viver com o HIV em todos os aspectos, tanto biológicos, psicológicos, sociais, culturais quanto espirituais, e sem restrição da localidade. Além disso, consideraram-se os estudos publicados na íntegra, sem restrição de idioma, limite temporal e delineamento metodológico. Também foram elegíveis artigos publicados em periódicos e na literatura cinzenta.

### Estratégia de Busca

A partir da busca preliminar na *Medical Literature and Retrieval System online* (MEDLINE) via *National Center for Biotechnology Information* (NCBI/ PubMed) e *Web of Science,* foram consideradas as palavras do texto contidas nos títulos e resumos dos artigos relevantes para desenvolver a estratégia de busca completa. Na sequência, foi realizado um piloto da busca final conduzido em duas bases de dados, a saber, MEDLINE via NCBI/PubMed e EMBASE. Após estas etapas, foi realizada a busca definitiva e, para cada base de dados e/ou fonte de informação incluída, a estratégia de busca, incluindo os termos índice identificados e palavras-chave, foram adaptadas. As buscas foram realizadas nas bases de dados: *Web of Science* (WOS), MEDLINE via NCBI/PubMed, *Science Direct,* Scopus, EMBASE via *Elsevier*, *Scientific Electronic Library Online* (SciELO), COCHRANE e a Literatura Latino-Americana e do Caribe em Ciências da Saúde (LILACS); Índice Bibliográfico Español en Ciencias de la Salud (IBECS); Base de Dados em Enfermagem (BDENF); Index Medicus para o Pacífico Ocidental (WPRIM); Literatura Peruana en Ciencias de la Salud (LIPECS); *World Health Organization’s Institutional Repository for Information Sharing* (WHO IRIS); Bibliografia Brasileira de Odontologia (BBO) via Biblioteca Virtual em Saúde (BVS), sendo acessadas por meio do Portal de Periódicos da Coordenação de Aperfeiçoamento de Pessoal de Nível Superior (CAPES). Foram consideradas como estratégias adicionais a busca em fontes de literatura cinzenta: Cybertesis, PeerJ Prepint, MedRxiv, OpenGrey, Preprints bioRxiv, Catálogo de teses e dissertações, e a busca de referências cruzadas. As buscas foram conduzidas entre julho e setembro de 2024.

Para a construção da estratégia de busca foram utilizados os operadores booleanos AND e OR, associados aos descritores MeSH (*Medical Subject Headings*), DeCS (Descritores em Ciências da Saúde), Emtree e descritores não controlados (Quadro 1).

**Quadro 1:** Construção, descritores/palavras-chaves e operadores booleanos utilizados na base de dados – Maringá, PR, Brasil, 2024.

Base de Dados	Estratégia de Busca
*Web of Science*	ALL = ((((((“Adolescent”) OR (“Young Adult”)) AND ((“adherence to antiretroviral therapy”) OR (“Medication Adherence”) OR (“Cooperation and Adherence to Treatment”) OR (“Patient Cooperation”) OR (“antiretroviral therapy highly active”)) AND ((“HIV Infections”) OR (“HIV”) OR (“Acquired Immunodeficiency Syndrome”))))))
MEDLINE/PubMed	(((“Adolescent” OR “Young Adult”) AND (“adherence to antiretroviral therapy” OR “Medication Adherence” OR “Cooperation and Adherence to Treatment” OR “Patient Cooperation” OR “antiretroviral therapy highly active”) AND (“HIV Infections” OR “HIV” OR “Acquired Immunodeficiency Syndrome”)
*Science Direct*	(“Adolescent” OR “Young Adult”) AND (“adherence to antiretroviral therapy” OR “Medication Adherence” OR “Cooperation and Adherence to Treatment” OR “Patient Cooperation” OR “antiretroviral therapy highly active”) AND (“HIV” OR “Acquired Immunodeficiency Syndrome”)
*Scopus*	((“Adolescent”) OR (“Young Adult”) AND (“adherence to antiretroviral therapy”) OR (“Medication Adherence”) OR (“Cooperation and Adherence to Treatment”) OR (“Patient Cooperation”) OR (“antiretroviral therapy highly active”) AND (“HIV Infections”) OR (“HIV”) OR (“Acquired Immunodeficiency Syndrome”))
*Embase*	(“Adolescent” OR “Young Adult”) AND (“adherence to antiretroviral therapy” OR “Medication Adherence” OR “Cooperation and Adherence to Treatment” OR “Patient Cooperation” OR “antiretroviral therapy highly active”) AND (“HIV Infections” OR “HIV” OR “Acquired Immunodeficiency Syndrome”)
*Scielo*	((((((“Adolescent”) OR (“Young Adult”)) AND ((“adherence to antiretroviral therapy”) OR (“Medication Adherence”) OR (“Cooperation and Adherence to Treatment”) OR (“Patient Cooperation”) OR (“antiretroviral therapy highly active”)) AND ((“HIV Infections”) OR (“HIV”) OR (“Acquired Immunodeficiency Syndrome”))))))
COCHRANE	(“Adolescent” OR “Young Adult”) AND (“adherence to antiretroviral therapy” OR “Medication Adherence” OR “Cooperation and Adherence to Treatment” OR “Patient Cooperation” OR “antiretroviral therapy highly active”) AND (“HIV Infections” OR “HIV” OR “Acquired Immunodeficiency Syndrome”)
LILACS; IBECS; BDENF; WPRIM; LIPECS; WHO IRIS; BBO via BVS	(((((((“Adolescent”) OR (“Young Adult”)) AND ((“adherence to antiretroviral therapy”) OR (“Medication Adherence”) OR (“Cooperation and Adherence to Treatment”) OR (“Patient Cooperation”) OR (“antiretroviral therapy highly active”)) AND ((“HIV Infections”) OR (“HIV”) OR (“Acquired Immunodeficiency Syndrome”))))))) AND (db:(“LILACS” OR “IBECS” OR “BDENF” OR “WPRIM” OR “LIPECS” OR “WHOLIS” OR “BBO”))
*Cybertesis*	(“Adolescent” OR “Young Adult”) AND (“adherence to antiretroviral therapy” OR “Medication Adherence” OR “Cooperation and Adherence to Treatment” OR “Patient Cooperation” OR “antiretroviral therapy highly active”) AND (“HIV Infections” OR “HIV” OR “Acquired Immunodeficiency Syndrome”)
*PeerJ Prepint*	(“Adolescent” OR “Young Adult”) AND (“adherence to antiretroviral therapy” OR “Medication Adherence” OR “Cooperation and Adherence to Treatment” OR “Patient Cooperation” OR “antiretroviral therapy highly active”) AND (“HIV Infections” OR “HIV” OR “Acquired Immunodeficiency Syndrome”)
*MedRxiv*	(“Adolescent” OR “Young Adult”) AND (“adherence to antiretroviral therapy”) AND (“HIV” OR “Acquired Immunodeficiency Syndrome”)
*OpenGrey*	(“Adolescent” OR “Young Adult”) AND (“adherence to antiretroviral therapy” OR “Medication Adherence” OR “Cooperation and Adherence to Treatment” OR “Patient Cooperation” OR “antiretroviral therapy highly active”) AND (“HIV Infections” OR “HIV” OR “Acquired Immunodeficiency Syndrome”)
*Preprints bioRxiv*	(“Adolescent” OR “Young Adult”) AND (“adherence to antiretroviral therapy”) AND (“HIV” OR “Acquired Immunodeficiency Syndrome”)
Catálogo de teses e dissertações	(“Adolescent” OR “Young Adult”) AND (“adherence to antiretroviral therapy”) AND (“HIV” OR “Acquired Immunodeficiency Syndrome”)

### Seleção das Fontes

Após as buscas nas fontes de informações, os resultados foram exportados para o aplicativo *Rayyan*® do *Qatar Computing Research Institute* (QCRI). Desse modo, das 10.540 publicações, houve 1.760 excluídas por duplicidade. Na sequência, 8.779 estudos foram para análise, sendo selecionados por título e resumo por dois revisores independentes com a finalidade de manter o processo de cegamento; posteriormente, foram comparados os estudos selecionados e chegou-se a um consenso. Não houve necessidade da atuação de um terceiro revisor. Após isso, 8.708 foram excluídos por não se enquadrarem nos critérios de inclusão.

Durante a leitura na íntegra, realizou-se uma leitura profunda aplicando os critérios de elegibilidade. As informações dos estudos foram organizadas e armazenadas em planilhas eletrônicas para garantir uma melhor interpretação, comparação entre os revisores.

Os artigos incluídos na amostra final foram organizados em um instrumento no *Microsoft Excel*®, sendo este adaptado em conformidade com a metodologia JBI^(8)^, no qual constavam os dados de caracterização das publicações (autores, ano, título, idioma, país, fonte de informação, método, medida de adesão, população, principais resultados).

### Análise, Extração e Apresentação dos Dados

Foi realizada uma leitura criteriosa para classificação dos textos e, na sequência, extraídos os resultados. Além disso, ocorreu uma busca manual para verificação da lista de referências dos estudos incluídos na revisão, o que resultou na inclusão de mais dez artigos. Esta etapa tinha como finalidade encontrar estudos que não estavam vinculados às bases de dados. Ao final, fizeram parte do presente estudo 39 publicações. A Figura 1 permite a visualização do processo de busca, exclusão e de seleção das publicações de acordo com o PRISMA^(9)^.

**Figura 1:** Fluxograma de identificação e seleção de artigos incluídos na revisão de escopo. Maringá, PR, Brasil, 2024.

Fonte: adaptação PRISMA-ScR Checklist^(9)^.

Todas as informações dos estudos incluídos foram armazenadas em planilhas e quadros analíticos. Os estudos incluídos foram analisados à luz dos Determinantes Sociais da Saúde^(11)^, o que facilitou o processo de interpretação e comparação das produções, consequentemente auxiliando na descrição das evidências e mapeamento de dados, os quais relacionaram-se à descrição dos determinantes sociais da adesão entre adolescentes e jovens vivendo com HIV. Os determinantes foram apresentados sob a forma de quadro.

Considerando que este estudo trata de uma revisão de escopo que utilizou publicações já disponíveis, não se faz necessária a apreciação por Comitê de Ética em Pesquisa. No entanto, a pesquisa foi desenvolvida considerando os aspectos éticos referentes à autoria dos artigos selecionados.

## RESULTADOS

A aplicação das estratégias de busca nas fontes de informação permitiu a identificação de 10.540 publicações. Durante a seleção de título e resumo, obtiveram-se 71 estudos para análise na íntegra, e desses, após aplicar os critérios de elegibilidade, 39 estudos foram selecionados, cujos resultados respondiam à questão norteadora ^(12–50)^.

### Características Gerais dos Estudos Incluídos

Observou-se que 37 (94,87%) ^(12–21,23–50)^ apresentavam o texto disponível no inglês, um (2,56%) no francês^(22)^ e um (2,56%) em português^(18)^. O período de publicações abrangeu os anos de 1999 a 2024, sendo 20 publicações relativas ao período 2017–2024 ^(31–50)^. Dos 39 estudos, houve 17 que relataram ter sido desenvolvidos no continente Africano: cinco em Uganda ^(24,30,39,45–46)^, três na África do Sul ^(42,44,50)^, dois em Botswana ^(21,37)^, Quênia ^(36,40)^, Etiópia ^(47,48)^ e Zâmbia ^(35,36)^, um em cada dos seguintes: Zimbábue^(27)^, África Subsaariana^(28)^, Malawi^(32)^, Camarão^(41)^, Nigéria^(43)^ e Essuatíni^(49)^; na América do Norte, foram desenvolvidos 15 estudos, sendo todos dos Estados Unidos da América ^(12–17,19–20,23,25,26,29,31,33,38)^; na América Latina foi elaborado apenas um estudo no Brasil^(18)^; e na Europa foi encontrada uma publicação proveniente da Bélgica^(22)^.

### Objetivos, Métodos, Medidas de Adesão e Participantes dos Estudos Incluídos

Em relação aos objetivos dos estudos, a maioria das publicações foi projetada para determinar os fatores associados à adesão ao tratamento do HIV entre adolescentes e jovens, em relação aos resultados promotores e limitantes da continuidade do seguimento. Já outras publicações se concentraram na avaliação da adesão, assim como nas experiências dos pacientes referentes às barreiras e aos facilitadores para adesão.

Referente aos métodos utilizados nos estudos, notou-se que 28 (71,79%) são quantitativos ^(12–17,19–23,26–30,32,34,38–41,44,46,47)^, sendo 23 com delineamento transversal ^(12–16,20–23,26–30,32,34,38–42,44,46,47,49,50)^, três coortes prospectivas ^(17,19,50)^, uma coorte retrospectiva^(42)^ e um longitudinal retrospectivo^(49)^; cinco (12,82%) qualitativos ^(18,24,31,37,45)^, sendo um do tipo fenomenológico^(45)^ e quatro descritivos ^(18,24,31,37)^; três (7,69%) métodos mistos ^(35,36,43)^, sendo que apenas um categorizado como paralelo convergente^(24)^, duas (5,12%) revisões sistemáticas com metanálise ^(25,48)^ e um (2,58%) de abordagem quantitativa e qualitativa^(33)^.

Em relação às medidas de adesão, 51 formas foram mencionadas, sendo 35 (68,62%) de medicação subjetiva voltadas a adesão autorrelatada ^(12,14–17,19–46,48,50)^, nove (17,64%) de métodos fisiológicos ^(14, 18, 22, 23, 25, 33, 42, 49, 50)^ e sete (13,72%) de medições farmacológicas ^(13,21,24,25,45,48,50)^. Dos 39 estudos, observou-se que 28 (71,80%) usaram apenas uma medida de adesão ^(12–17,19–21,26,27,29–32,34–40,43–47,50)^, oito (20,51%) usaram duas medicações ^(18,22–24,41,42,48,49)^ e três (7,69%) estudos utilizaram três medidas de adesão ^(25,28,33)^. Com relação aos participantes, houve apenas um estudo realizado com adolescentes e jovens junto aos seus familiares^(33)^. Referente à faixa etária dos participantes, notou-se que 15 (38,47%) estudos englobavam adolescentes e jovens (10–24 anos) ^(12,13,16, 18–20,22,23,26,29,31,38,43,45,47)^ e 24 (61,53%) somente adolescentes (10–19 anos) ^(14,15,17,21,24,25,27,28,30–37,39–42,44,46,48,49,50)^.

### Estrutura Conceitual

Os fatores promotores e limitantes identificados nos estudos incluídos foram organizados em cinco dimensões relacionadas aos determinantes que influenciam na adesão ao tratamento entre adolescentes e jovens vivendo com HIV: Dimensão 1 – Características Individuais; Dimensão 2 – Estilo de vida e comportamentos individuais; Dimensão 3 – Redes sociais e comunitárias; Dimensão 4 – Condições de vida e de saúde; Dimensão 5 – Condições socioeconômicas, culturais e ambientais. Os determinantes foram resumidos no Quadro 2.

**Quadro 2:** Resumo dos determinantes identificados nos estudos incluídos (n = 39) na revisão de escopo. Maringá, PR, Brasil, 2024.

Dimensão dos determinantes	Determinantes sociais da adesão à terapia antirretroviral de adolescentes e jovens vivendo com HIV	
	Promotores	Limitantes
Dimensão 1: Características Individuais	**1. Compreensão da saúde e tratamento:** Maior alfabetização em saúde^(23)^ Conhecimento do estado sorológico ^(25,48)^ Compreensão da doença^(26)^ Compreensão da relevância da medicação^(31)^ Conhecimento da eficácia da TARV^(36)^ Utilidade percebida dos medicamentos para o HIV^(45)^ **2. Motivação e autoeficácia:** Esperam prolongar sua vida por pelo menos 10 anos^(12)^ Motivadores orientados para objetivos pessoais^(31)^ Desejo de ser saudável e viver^(37)^ Prontidão motivacional para a TARV^(38)^ Maior autoeficácia ^(38,40)^ Maneiras de se automotivar para atingir a adesão^(43)^ **3. Estratégias de gestão do tratamento:** Uso de auxiliares de memória^(31)^ Uso de lembretes^(43)^ Estratégias de ocultação^(43)^ Sabem instintivamente quando é hora de usar seus antirretrovirais^(43)^ **4. Atitudes e crenças pessoais:** Medo das consequências negativas da não adesão^(34)^ Satisfação com a vida^(46)^	**1. Fatores relacionados à idade e desenvolvimento:** Jovens com idade mais avançada ^(17,33,42,46)^ Crescimento atrofiado^(25)^ **2. Esquecimento e falta de Consistência:** Esquecimento ^(20,22,32–34,37)^ Adolescente nunca leva a TARV enquanto está fora^(25)^ Ter perdido doses de TARV por falha em retirar na farmácia^(21)^ **3. Falta de motivação e recusa da TARV:** Não sentir vontade de tomar medicamentos^(20)^ Não querer ser lembrado da infecção pelo HIV^(20)^ Baixa autoeficácia no tratamento^(32)^ Adolescente não se preocupa com a TARV^(25)^ **4. Fatores contextuais:** Sexo masculino ^(21,25,46)^ Sexo feminino^(47)^ Ser órfão duplo^(25)^ Falta de conhecimento básico sobre HIV^(35)^ Adolescentes com HIV adquirido horizontalmente^(42)^
Dimensão 2: Estilo de vida e comportamentos individuais	**1. Estabilidade e rotina de Vida:** Estabilidade das situações de vida^(13)^ Estilo de vida e existência de uma rotina diária^(31)^ Adolescentes nas trajetórias de adesão com início baixo e crescente^(50)^ **2. Saúde mental e bem-estar:** Bem-estar e baixo sofrimento psicológico ^(29,38,44)^ Menor uso de álcool ^(17,29)^	**1. Problemas de saúde mental:** Depressão ^(14,16,17,19,24,35)^ Sofrimento psicológico ^(20,29)^ Baixa pontuação de saúde mental^(25)^ Aumento unitário no sofrimento psicológico^(28)^ Problemas emocionais^(31)^ Sentir-se deprimido/sobrecarregado^(32)^ Infelicidade autorrelatada^(33)^ **2. Uso de Substâncias:** Uso de substâncias ^(12,20,38)^ Uso de maconha ^(16,29)^ Uso de álcool^(32)^ **3. Comportamentos e atividades distrativas:** Estar ocupado com outras coisas^(32)^ Ter namorado/namorada, bate-papo on-line frequente^(33)^ **4. Comportamentos de não-adesão:** Recusa intencional de tomar a medicação^(43)^ Ser sexualmente ativo^(25)^
Dimensão 3: Redes sociais e comunitárias	**1. Apoio Familiar:** Apoio dos familiares^(18)^ Administração do medicamento pelos pais^(25)^ Coesão familiar^(39)^ Comunicação adolescente-cuidador^(39)^ Revelaram seu status de HIV (mãe)^(12)^ Ter cuidador com companheiro^(25)^ Ter o responsável presente em cada encontro clínico^(27)^ **2. Apoio pelo profissional de saúde:** Apoio da equipe de saúde^(18)^ Profissionais de saúde que prestam apoio^(24)^ Adolescentes que foram ensinados a tomar TARV por um profissional de saúde^(25)^ Conforto em fazer perguntas ao profissional de saúde^(27)^ **3. Apoio social entre pares e grupos:** Apoio de pares^(24)^ Aconselhamento dos profissionais de saúde^(24)^ Apoio social e emocional e o aconselhamento do grupo de pares^(33)^ Apoio de parceiros de tratamento ou família^(43)^ Apoio social e serviços de aconselhamento^(36)^ Recursos comunitários para o HIV^(36)^ Maior apoio social ^(38, 45)^ **4. Estratégias de Enfrentamento e Recursos:** *Coping* (estratégias de enfrentamento proativas; recorrer à família; enfrentamento espiritual; ajuda profissional)^(19)^ Apoio no ambiente domiciliar^(37)^ Não tomar medicamento na escola^(17)^ Adolescente não tem conhecimento dos problemas de saúde do cuidador^(25)^	**1. Características do cuidador:** Cuidador sendo viúvo^(25)^ Cuidador não tendo uma prática religiosa^(25)^ Baixo envolvimento do cuidador^(25)^ Baixo nível de educação do cuidador^(25)^ Baixa capacidade intelectual avaliada pelo cuidador^(33)^ Ter avós ou parentes como cuidador principal^(33)^ Ter um avô como cuidador principal^(46)^ Cuidador sendo o único que conhece o status sorológico da criança^(25)^ **2. Dinâmica familiar e relacional prejudicada:** Ter família desorganizada e arranjos in loco *parentis* ^(25)^ Relações tensas com os cuidadores^(33)^ Falta de cuidado, apoio e amor do pai^(43)^ Culpar quem contaminou a mãe^(43)^ **3. Violência e trauma:** Testemunhar ou sofrer violência em casa^(32)^ Perda da mãe^(35)^ **4. Comunicação e Colaboração:** Baixa comunicação com o médico^(33)^ Falta de colaboração entre organizações e normas sociais^(36)^
Dimensão 4: Condições de vida e de saúde	**1. Aspectos clínicos do tratamento:** Nível de CD4 ≥ 500 células/mm3^(13)^ Aumento das contagens de CD4+^(34)^ Cargas virais <1000 cópias/ml^(40)^ Carga viral basal indetectável^(49)^ Tomando profilaxia com cotrimoxazol em conjunto com a TARV^(25)^ Número de medicamentos prescritos^(13)^ Ausência de efeitos colaterais e nutrição/dieta^(31)^ Privacidade para tomar o medicamento^(31)^ Características dos medicamentos (número e tamanho dos comprimidos, ao regime)^(31)^ Uma vez por dia, a dosagem^(40)^ **2. Recursos para condições de vida e saúde:** Participar de sessões de grupo lideradas por um facilitador profissional^(27)^ Fornecimento de alimentos^(24)^ Comida suficiente para comer enquanto tomavam a medicação^(44)^ Suprimento adequado de medicamentos^(36)^ Aqueles que tiveram atendimento para sua preocupação dentro da unidade^(47)^ **3. Acessibilidade ao serviço de saúde:** Curto tempo de espera^(24)^ Viver mais perto de uma clínica^(30)^ Disponibilidade/acessibilidade (ter transporte) ^(24,31)^ Dinheiro suficiente para viajar até à clínica^(44)^ Viagens perto do domicílio^(32)^ Disponibilidade de serviços gratuitos^(45)^	**1. Condições de moradia e alimentação prejudicada:** Instabilidade ou falta de moradia ^(12,13)^ Falta de nutrição adequada^(36)^ **2. Aspectos relacionados ao tratamento:** Muitos comprimidos ^(12,24)^ Tomar 3 ou mais comprimidos por dia^(25)^ Dificuldade de administração do medicamento pelo cuidador^(25)^ Medicamento administrado pelo adolescente^(25)^ Adolescentes na fase mais avançada do HIV, apresentando aids^(17)^ Histórico anterior de infecção oportunista^(47)^ Carga viral de RNA ≥1000 cópias/ml^(33)^ Carga viral basal detectável^(49)^ Não seguir o regime prescrito^(28)^ **3. Efeitos colaterais e carga do tratamento:** Efeitos adversos relacionados à medicação ^(15,24,35,41,43)^ Intolerância à medicação^(25)^ Fadiga medicamentosa ^(24,31)^ Cansaço de tomar os medicamentos todos os dias pelo resto da vida^(43)^ Carga percebida do tratamento^(45)^ Efeito percebido da TARV na aparência física^(43)^ **4. Baixo acesso e suporte do serviço de saúde:** Morar longe do serviço, seja área urbana ou rural^(25)^ Viver em áreas distantes^(50)^ Baixa satisfação com os cuidados de saúde^(19)^ Falta de serviços específicos para adolescentes^(36)^ Cuidador não paga uma taxa pelo tratamento^(25)^ Complicações nas rotinas do dia a dia^(15)^ Faltar à consulta médica nos últimos 6 meses^(32)^ Falta a uma ou mais doses de TARV na última semana^(36)^ Longo tempo de espera na clínica^(34)^
Dimensão 5: Condições socioeconômicas, culturais e ambientais	**1. Recursos econômicos:** Maior posse de bens, especificamente a posse familiar de sete ou mais bens tangíveis^(30)^ Maior vantagem econômica na posse de bens domésticos, poupanças financeiras e emprego de cuidador^(30)^ Melhorias dentro e entre pessoas no bem-estar econômico^(44)^ **2. Redução do Estigma:** Redução do estigma internalizado do HIV^(44)^	**1. Estigma e discriminação:** Preconceito, discriminação e estigma ^(18,24,34,36,37)^ Estigma internalizado^(41)^ Sentir-se estigmatizado por pessoas de fora e dentro de casa^(32)^ Estigma e discriminação resultantes percebidos^(45)^ **2. Medos relacionados à revelação:** Medo de ser visto tomando o antirretroviral ^(22,24)^ Medo da revelação ^(31,36,37,43)^ Medo de revelar o estado de HIV a outras pessoas, especialmente a rapazes/namoradas^(33)^ **3. Condições socioeconômicas:** Pobreza ^(24,36)^

Fonte: Dados da pesquisa, 2024.

Esta revisão identificou que os fatores voltados às características individuais, estilo de vida, redes sociais e comunitárias, condições de vida e trabalho, condições socioeconômicas, culturais e ambientais de adolescentes e jovens influenciaram na adesão ao tratamento de HIV, sendo moldados constantemente por serem multiníveis, ou seja, interagirem de forma complexa. Assim, os determinantes sociais da adesão afetam o contexto do tratamento antirretroviral, tanto de forma positiva quando negativa, e estão diretamente interligados às condições de vida dos adolescentes e jovens vivendo com HIV (Figura 2).

**Figura 2:** Síntese conceitual dos resultados advindos da amostra final dos determinantes sociais da adesão entre adolescentes e jovens vivendo com HIV. Maringá, Paraná, Brasil, 2024.

Fonte: Dados da pesquisa, 2024.

## DISCUSSÃO

Os estudos incluídos na revisão cobrem uma ampla gama de países e continentes, como América do Norte, América Latina, África, Ásia e Europa, sendo países de alta, média e baixa renda média per capita. As diversidades geográficas e culturais são importantes para entender como diferentes contextos sociais, econômicos e culturais influenciam na adesão à terapia antirretroviral entre adolescentes e jovens. A adaptação de estratégias de intervenção deve considerar essas variações contextuais^(51)^.

No que tange a temporalidade dos materiais encontrados, parte significativa dos estudos foram publicados entre os anos de 2017 e 2024. Este fato deve-se especialmente ao lançamento da Declaração Política da Assembleia Geral das Nações Unidas sobre o fim da aids, e a redução de novas infecções por HIV. A partir do lançamento desta declaração, em 2016, os países do mundo todo comprometeram-se a acelerar suas ações de luta contra o HIV e aids para acabar com a epidemia até o ano de 2030^(52)^.

Percebe-se que a minoria dos estudos foi de abordagem qualitativa, sendo que estes são essenciais para explorar as barreiras e facilitadores da adesão à TARV entre adolescentes e jovens. A abordagem qualitativa permite uma análise mais profunda das percepções, experiências e contextos sociais dos adolescentes e jovens vivendo com HIV, além de elucidar questões psicossociais e culturais que afetam a adesão, o que pode não ser captado por estudos quantitativos ^(53,54)^.

Em relação à dimensão das características individuais dos adolescentes e jovens vivendo com HIV, notou-se que o maior conhecimento do estado sorológico e da importância do antirretroviral são determinantes que promovem a adesão. Dessa forma, se o jovem tem acesso à informação e a compreende, isso o leva a ter conhecimento acerca do HIV/aids e da TARV. Essas evidências auxiliam os gestores nas decisões sobre capacitar e manter conselheiros e profissionais de saúde atualizados para que eles possam, por sua vez, transmitir informações precisas aos adolescentes e jovens^(55)^.

Assim, a alfabetização em saúde tem sido um fator que influencia na adesão ao tratamento de inúmeras infecções e doenças, em especial do HIV e aids. Os pacientes que recebem informações adequadas ao seu nível de compreensão possuem resultados significativos ao longo do tratamento ^(56,57)^. O conhecimento e as crenças sobre o HIV dos adolescentes e jovens vivendo com HIV desempenham um papel essencial em sua experiência^(58)^.

Atrelados a estes fatores individuais, os objetivos pessoais e o desejo de viver, somados à satisfação com a vida, são aspectos que promovem a adesão ao tratamento. Estudo transversal realizado na China apontou que as pessoas que aceitam o diagnóstico de HIV aderem ao tratamento e o realizam de maneira correta. Consequentemente, possuem maior qualidade e esperança de vida^(59)^. A aceitação do HIV como condição crônica ajuda a mudar o comportamento de adolescentes e jovens e, por sua vez, os leva a uma mudança de estilo de vida, com o objetivo de tornarem-se indetectáveis e intransmissíveis (I = I)^(58)^.

No entanto, algumas características pessoais prejudicam a realização de um tratamento correto, como jovens de idade mais avançada e ser do sexo masculino. Dessa forma, ser jovem já é um fator, uma vez que sua responsabilidade é vista como uma barreira, relacionada ao cansaço em realizar um tratamento contínuo, além da comparação com pessoas do mesmo círculo social e o envolvimento em outras atividades e costumes próprios da faixa etária. Outrossim, os jovens do sexo masculino têm mais dificuldade de buscar ajuda no serviço de saúde, devido ao próprio estereótipo de gênero, prejudicando o engajamento dos homens em toda a cascata do cuidado. Dessa forma, tratando-se de uma fase em que recebem menor apoio social e familiar, os adolescentes mais velhos e os jovens são mais propensos a não aderir ao tratamento ou ter uma má adesão^(57)^.

Ademais, uma revisão sistemática e metanálise realizada com adolescentes órfãos mostrou que o status de orfandade é uma barreira significativa para a adesão à TARV, especialmente em países de baixa renda. A dependência de cuidadores, que pode ser inconsistente ou insuficiente, agrava esse desafio, indicando a necessidade de intervenções que aumentem o apoio social e melhorem a educação dos cuidadores sobre a importância da adesão ao tratamento^(60)^.

Somado a isto, vemos que muitos adolescentes e jovens apresentam lapsos ocasionais em tomar seus medicamentos antirretrovirais devido ao esquecimento, que muitas vezes é causado pelo estresse psicológico de ter a infecção e/ou doença e ter que tomar o medicamento diariamente^(61)^.

Quanto à dimensão de estilo de vida e comportamentos individuais, percebeu-se que o bem-estar psicológico é um determinante importante que deve ser levado em consideração em pessoas que vivem com HIV, em especial adolescentes e jovens, pois é um dos fatores que contribuem para o sucesso do tratamento. Estes indivíduos dedicam-se ao tratamento com a finalidade de prolongar a vida e adotam um estilo de vida saudável, com uma rotina que minimiza o sofrimento psicológico, capaz de vivenciar situações de resiliência frente a condição^(62)^.

No entanto, os transtornos mentais e de sofrimento psicológico, como ansiedade e depressão, são corriqueiros na vida destes jovens, que somados ao abuso de substâncias lícitas e ilícitas, podem levar ao abandono do tratamento^(62)^. Dessa forma, vivenciar dissonância emocional e ter dificuldade em abraçar sua própria identidade mostram que a saúde mental está frágil, impactando no tratamento^(58)^.

Na dimensão redes sociais e comunitárias, percebeu-se que o apoio, seja ele social, familiar ou do profissional de saúde, é um determinante que contribui positivamente para a adesão à terapia antirretroviral entre adolescentes e jovens vivendo com HIV. As relações sociais são necessárias a todos os indivíduos, em especial para pessoas que vivem com uma doença crônica, em que a rede de apoio e as relações de confiança são importantes para o enfrentamento da doença^(63)^. Um estudo realizado com adolescentes peruanos destacou como os fatores sociais e culturais, incluindo o apoio familiar e as dinâmicas sociais, influenciam na adesão ao tratamento. O uso de modelos ecológicos sociais nesses estudos enfatiza a necessidade de considerar múltiplos níveis de influência, desde o nível individual até o comunitário, ao desenhar intervenções para melhorar a adesão à TARV^(53)^.

Além disso, as relações de confiança estabelecidas com profissionais de saúde são importantes para alguns pacientes, sendo, na maioria dos casos, a única fonte de apoio e orientação^(57)^. Uma revisão sistemática da literatura identificou que o aconselhamento e a educação sobre a terapia antirretroviral são facilitadores da adesão ao tratamento, ao considerar o paciente em sua totalidade, incluindo os aspectos étnicos, culturais, socioeconômicos e o nível educacional do indivíduo ^(56,57)^.

Nesse mesmo sentido, o apoio entre pares é importante para enfrentar o tratamento; no entanto, alguns jovens sentem-se envergonhados e diferentes quando se relacionam com parceiros que não tenham a mesma condição, não encontrando um apoio mútuo^(64)^. Dessa forma, alguns adolescentes e jovens que vivem com HIV buscam continuamente e podem ter recebido apoio de outras pessoas significativas e da comunidade em sua vida, fazendo com que o tratamento e o diagnóstico se tornem algo mais leve em seu dia a dia^(58)^.

A dinâmica familiar prejudicada, e o relacionamento de um jovem que vive com HIV com os demais membros da família e os traumas no ambiente domiciliar ainda são determinantes que têm influenciado de forma negativa a adesão à terapia antirretroviral. O estigma e preconceito, na maioria dos casos, parte da própria família, e corroboram sintomas depressivos e ideações suicidas^(57)^.

Assim, torna-se imprescindível investir no suporte social para esses pacientes junto aos seus familiares que sabem do diagnóstico e a equipe de saúde, para que se possa melhorar a qualidade de vida e sobrevida deles a partir da adesão à TARV^(65)^. Além disso, destaca-se que apenas um estudo^(33)^ trouxe a perspectiva do cuidador familiar. Este resultado remete à necessidade de desenvolvimento de novos estudos nesse âmbito com a finalidade de melhorar a adesão à terapia antirretroviral junto com o apoio dos familiares de adolescentes e jovens vivendo com HIV.

Percebe-se que na dimensão condições de vida e de saúde, os determinantes promotores estão relacionados ao tratamento e à acessibilidade aos serviços de saúde. É possível identificar que a carga viral <1000 cópias/ml ou indetectável e um nível da contagem de CD4+ ≥ 500 células/mm3 ou aumentada está associada a adesão, uma vez que os jovens se sentem incentivados a continuar o tratamento. A supressão do HIV é uma estratégia essencial para otimizar a saúde e o bem-estar daqueles que vivem com a infecção. Os testes de rotina de carga viral (CV) e contagem de CD4+ são essenciais para melhorar as oportunidades de tratamento e são uma ferramenta particularmente útil entre grupos com elevado risco de falência virológica, como os adolescentes^(66)^.

Estudo realizado no Reino Unido e na Irlanda mostrou que nos jovens que iniciaram a TARV antes dos 10 anos, a contagem de CD4+ diminuiu a partir dos 10 anos e que a supressão viral <400 cópias/ml ao longo do tempo apresentou contagens médias de CD4+ que se aproximavam da imunodeficiência leve (350–500 células/mm3) aos 20 anos^(67)^.

Os efeitos adversos dos antirretrovirais foi considerado um limitador da adesão à TARV, uma vez que adolescentes e jovens vivendo com HIV frequentemente lutam com o autogerenciamento do tratamento. Muitos apresentam sintomas devido à doença do HIV, condições comórbidas e/ou efeitos adversos dos antirretrovirais^(62)^.

Os efeitos adversos implicam diretamente na adesão, pois afetam o dia a dia do adolescente e/ou do jovem e consequentemente sua qualidade de vida. Assim, para que se possa neutralizar as barreiras e, ao mesmo tempo, melhorar os facilitadores, a otimização do tratamento com regimes baseados em dolutegravir (DTG), abordagens orientadas por pares, como os apoiadores de pares jovens e adolescentes e modelos diferenciados de prestação de serviços, incluindo dispensação de vários meses é uma excelente estratégia para minimizar o impacto do tratamento na vida deles ^(68–70)^.

A distância geográfica tem sido preponderante na adesão ao tratamento e cuidado em HIV, principalmente devido à sua cronicidade. Em sua maioria é um fator que limita o acesso aos serviços de saúde, sendo uma barreira que interfere de forma direta nas taxas de adesão e abandono do tratamento quando são distâncias maiores ^(65,71)^. A falta de recursos financeiros também reflete no acesso ao tratamento e, somada à distância pode interferir tanto nos desfechos da própria saúde do indivíduo, quanto também na dinâmica dos serviços, pois estes acabam faltando nas consultas e rotinas ou abandonando o tratamento ^(65,72)^.

De igual modo, a acessibilidade aos serviços de saúde associada à distância de residência, e aos fatores socioeconômicos dos indivíduos são barreiras para uma boa adesão à TARV, potencializando a vulnerabilidade dos adolescentes^(57)^. Em contrapartida, quando os adolescentes e jovens moram perto do serviço de HIV, acabam buscando o serviço com mais frequência, justamente pelo fato de não precisarem pagar pelo atendimento e por residirem próximos ao local de consulta e retirada de medicação ^(30,45)^.

Assim, torna-se importante que o serviço especializado de HIV seja integrado aos demais níveis de serviços de saúde, em especial aqueles que compõem a atenção primária, pois a falta de integração tem dificultado o desenvolvimento de estratégias e ações de prevenção e promoção para essa população^(71)^.

Posto isto, torna-se essencial que os serviços de saúde voltados ao HIV também realizem o monitoramento das ausências e seus motivos, e assim, procurem alternativas para maior adesão às consultas de acompanhamento e ao uso da TARV. Além disso, deve-se conscientizar os pacientes para que possam ter responsabilidade pela própria saúde, de modo a se envolver no cuidado e, consequentemente, melhorar sua qualidade de vida^(73)^.

Já a dimensão das condições socioeconômicas, culturais e ambientais trazem à tona a necessidade de a sociedade e os adolescentes e jovens vivendo com HIV reduzirem os estigmas. Estudo realizado nas Filipinas mostrou que lidar com estereótipos sobre o HIV contribuiu para a maneira como adolescentes e jovens assumem os julgamentos de outros (estigma percebido) e assim internalizam o estigma que altera sua crença em relação a si mesmos e à doença^(58)^. Ainda se percebe também que o estigma e a discriminação são causados por desinformação sobre a doença e decorrem tanto de si mesmos quanto de outras pessoas ao seu redor^(58)^.

Há evidências de que o preconceito, a discriminação, o estigma e o medo da revelação do diagnóstico têm prejudicado significativamente a adesão à terapia antirretroviral. Estudo realizado com adolescentes na Etiópia ressaltou que as barreiras do estigma, preconceito, discriminação e medo de expor o diagnóstico têm sido as mais prevalentes. Assim, a pesquisa instiga intervenções direcionadas a melhorar o suporte social e reduzir o estigma, preconceito e discriminação, fazendo que com dessa forma os adolescentes e jovens deixem o medo de lado e possam conviver melhor com o HIV^(74)^.

Estudos qualitativos recentes, como os realizados em países da África Subsaariana e no Peru ^(53,54)^, destacam o papel crítico do estigma, do medo de divulgação e dos desafios emocionais enfrentados pelos adolescentes, sendo estes determinantes para a adesão ótima à TARV. Destaca-se que a pandemia de COVID-19 exacerbou essas barreiras, aumentando o isolamento social e dificultando o acesso aos serviços de saúde, o que impactou negativamente na adesão à TARV em várias regiões do mundo^(75)^.

A pobreza também tem sido um fenômeno profundamente estigmatizado devido aos aspectos de desigualdade social, além de ser fator que contribui para a insegurança alimentar e habitacional, repercutindo negativamente na adesão ao tratamento. Dessa forma, torna-se essencial lidar com a insegurança de recursos para que se possa chegar ao cerne do estigma relacionado ao HIV^(76)^.

Os adolescentes e jovens vivendo com HIV em países de baixa e média renda apresentam diferentes contextos geográficos e socioeconômicos; consequentemente, têm um risco maior de problemas psicossociais, de desenvolvimento e comorbidades, tornando-os um grupo particularmente vulnerável à adesão ao tratamento. Além disso, há também discrepâncias entre cenários de recursos, a falta de estrutura e a comunicação ineficaz entre os provedores de cuidados de saúde^(77)^.

Todas essas dimensões têm implicações para as estratégias de promoção da saúde pública no contexto da adesão ao tratamento de adolescentes e jovens vivendo com HIV, incluindo a concepção e desenvolvimento de intervenções baseadas no agregado familiar, social e de profissionais de saúde. As estratégias eficazes devem ser multifacetadas, abordando os fatores comportamentais, sociais e estruturais para minimizar os casos de má-adesão e perda de seguimento.

Nesse âmbito encontram-se os esforços internacionais, com o objetivo de acabar com o HIV como problema de saúde pública até 2030, com a proposta de que as desigualdades sociais sejam combatidas e ações intersetoriais sejam ampliadas. Alcançar a meta 95-95-95 deve ser o alvo de todos os países do mundo, e para isso é necessária disponibilidade de testagem e tratamento em tempo oportuno somados à remissão da carga viral. Assim como a adesão à terapia medicamentosa, também se faz necessário que os adolescentes e jovens que vivem com HIV tenham uma postura de autocuidado ativo, por meio da adoção de modo de vida saudável, comparecendo às consultas, realizando os exames, retirando os medicamentos na data prevista, bem como tomando o medicamento diariamente de forma correta. Dessa forma, o centro do cuidado são os adolescentes e jovens vivendo com HIV; logo, os profissionais de saúde devem estimular a postura ativa no próprio tratamento^(78)^.

### Limitações do Estudo

Como limitação, destaca-se o fato de se ter considerado todos os tipos de medidas de adesão, o que implica em possíveis vieses da amostra como também na heterogeneidade metodológica associada a cada tipo de medida, impossibilitando a generalização dos dados. Outro limitante é a não inclusão de textos em outras bases de indexação; dessa forma, reconhece-se que importantes pesquisas publicadas podem ter sido omitidas com a estratégia de busca utilizada nesta revisão. Entretanto, salienta-se que todos os rigores científicos exigidos para uma revisão de escopo foram seguidos de acordo com as recomendações do JBI. Além disso, os resultados da síntese conceitual devem ser interpretados com cautela, uma vez que apresentam diferentes cenários epidemiológicos e socioeconômicos, assim como medidas e estratégias implementadas, aspectos operacionais e organizacionais, e o formato do sistema de saúde em cada país.

### Avanços Para a Área da Enfermagem e da Saúde

Os profissionais de saúde, em especial os enfermeiros que atuam nos ambulatórios de HIV e na Atenção Primária à Saúde (APS) atendendo adolescentes e jovens, podem se beneficiar dos resultados, uma vez que o marco conceitual sintetizado traz à tona situações do cotidiano e que influenciam de forma direta no tratamento. Logo, com determinantes promotores e limitantes sendo reconhecidos, o processo de busca por estratégias faz com que a diminuição das taxas de má-adesão e não-adesão do tratamento se torne menos árdua e mais específica. A partir desse estudo, novas pesquisas podem ser realizadas a fim de eliminar os fatores que sabidamente limitam o tratamento de adolescentes e jovens vivendo com HIV em várias frentes e em diferentes contextos.

## CONCLUSÃO

O mapeamento dos determinantes que promovem e/ou limitam a adesão à terapia antirretroviral entre adolescentes e jovens vivendo com HIV possibilitou mitigar a (re)estruturação das políticas públicas em relação à prática clínica, especialmente em serviços de saúde que atendem as necessidades desse público, na redução das desigualdades sociais e na promoção da adesão ao tratamento, consequentemente levando ao cumprimento das metas até 2030. As descobertas sintetizadas destacam que a adesão ao tratamento está alicerçada nas crenças e saberes pessoais, na dinâmica da rotina no ambiente familiar e no acesso aos serviços de saúde. Este estudo também suscita a necessidade de realização de novas pesquisas que busquem capturar a diversidade de fatores que refletem na experiência vivida e comportamentos relacionados à adesão do tratamento antirretroviral no contexto da adolescência e juventude.

## DISPONIBILIDADE DE DADOS

O conjunto de dados que dá suporte aos resultados deste estudo não está disponível publicamente, no entanto, todos os dados que dá suporte aos resultados deste estudo está disponível mediante a solicitação à autora correspondente.

# Translation: Determinantes sociales de la adherencia a la terapia antirretroviral entre adolescentes y jóvenes que viven con el VIH: *revisión de alcance*

**DOI:** 10.1590/1980-220X-REEUSP-2025-0026es

**Authors:** Camila Moraes Garollo Piran, Mariana Martire Mori, Alana Vitória Escritori Cargnin, Beatriz Sousa da Fonseca, Bianca Machado Cruz Shibukawa, Maria de Fátima Garcia Lopes Merino, João Manuel Graça Frade, Marcela Demitto Furtado

**Affiliations:** 1 Universidade Estadual de Maringá, Programa de Pós-Graduação em Enfermagem, Maringá, PR, Brasil.; 2 Politécnico de Leiria, Escola Superior de Saúde, Leiria, Portugal.; 3 Universidade Federal de Mato Grosso do Sul, Departamento de Enfermagem, Três Lagoas, MS, Brasil.

**Keywords:** VIH, Adolescente, Adulto Joven, Cumplimiento de la Medicación, Determinantes Sociales de la Salud

## Abstract

**Objetivo:**

Mapear la evidencia científica relacionada con los determinantes sociales de la adherencia a la terapia antirretroviral entre adolescentes y jóvenes que viven con VIH.

**Método:**

Revisión del alcance según la metodología del JBI para la revisión del alcance, realizado en 20 bases de datos. Los criterios de inclusión fueron: adolescentes y jóvenes viviendo con VIH (10 y 24 años), estudios relacionados con la adherencia a la terapia antirretroviral (adherencia autoinformada, supresión viral, recuento de pastillas o registros de recarga de farmacia).

**Resultados:**

Se identificaron 39 estudios, publicados entre 1999 y 2024, que permitieron mapear los determinantes que promueven y limitan la adherencia al tratamiento entre adolescentes y jóvenes que viven con VIH, siendo centrado en las características individuales, estilo de vida, redes sociales y comunitarias, condiciones de vida y de trabajo, condiciones socioeconómicas, culturales y ambientales (Dimensiones 1 a 5).

**Conclusión:**

Los determinantes sociales de la adherencia interactúan de forma compleja, lo que afecta el contexto de la terapia antirretroviral, tanto positiva como negativamente, y están directamente interconectados con las condiciones de vida de los adolescentes y jóvenes que viven con VIH.

## INTRODUCCIÓN

En todo el mundo, los adolescentes y los jóvenes representan una proporción cada vez mayor de las personas que viven con el VIH. En 2022, había 480 000 adolescentes y jóvenes de entre 10 y 24 años recién infectados por el VIH, de los cuales 140 000 eran adolescentes de entre 10 y 19 años. Además, el 25% de las adolescentes y el 17% de los adolescentes de entre 15 y 19 años son de África Oriental y Meridional, las regiones más afectadas por el VIH, que se han sometido a la prueba en los últimos 12 meses y han recibido el resultado de la última prueba. Si se mantiene la tendencia actual, en 2030 habrá 183 000 nuevas infecciones anuales por VIH entre adolescentes y jóvenes^(1)^.

Se estima que la cobertura del tratamiento entre los adolescentes y jóvenes de entre 15 y 24 años que viven con el VIH fue del 55%, significativamente inferior a la cobertura del 75% entre los de más edad^(2)^. Por lo tanto, se destaca la necesidad de adherirse al tratamiento contra el VIH, ya que este puede salvar vidas cuando se inicia temprano y se utiliza adecuadamente. Sin embargo, se sabe que cada año varios adolescentes y jóvenes sucumben a complicaciones relacionadas con el sida como consecuencia de la mala adherencia o el abandono del tratamiento^(3)^.

Los adolescentes y jóvenes que viven con el VIH se enfrentan a la carga de los retos físicos, psicológicos y sociales propios de la adolescencia y la juventud, al tiempo que lidian con una enfermedad infecciosa estigmatizada que requiere cuidados durante toda la vida y, a menudo, no buscan asistencia en los servicios de salud^(4)^. Esto se refleja en resultados de salud negativos entre los adolescentes y jóvenes en comparación con otros grupos de edad^(5)^.

Por lo tanto, es necesario adaptar los servicios para los adolescentes y los jóvenes de manera equitativa, accesible, aceptable, adecuada y eficaz^(6)^. En consonancia con ello, parte de los Objetivos de Desarrollo Sostenible de las Naciones Unidas incluyen el fin de la epidemia del sida y la meta de alcanzar la cobertura universal de los servicios de salud sexual y reproductiva para 2030^(7)^. Para alcanzar estos objetivos, es necesario identificar los factores que facilitan y limitan la adherencia al tratamiento del VIH entre los adolescentes y los jóvenes, contribuyendo con los profesionales a elaborar un plan de atención individualizado e integral para cada paciente^(2)^.

Dicho esto, se necesitan nuevas iniciativas que combatan las barreras persistentes al acceso a los servicios de tratamiento entre los adolescentes y los jóvenes que viven con el VIH, y se deben crear estrategias para alcanzar los objetivos globales. Aunque existe bibliografía sobre la adherencia al tratamiento entre este público, no se evidencian los determinantes sociales de la adherencia que promueven y limitan la continuidad del tratamiento, así como el acceso al servicio de salud, lo que constituye una laguna de conocimiento que debe explorarse. En este sentido, el objetivo de este estudio fue mapear la evidencia científica relacionada con los determinantes sociales de la adherencia a la terapia antirretroviral entre adolescentes y jóvenes que viven con el VIH.

## MÉTODO

### Tipo de Estudio

Se trata de una *revisión exploratoria* de acuerdo con la metodología JBI para la revisión del alcance, cuyo objetivo es mapear la literatura actual, así como enumerar los principales conceptos del área y las lagunas de conocimiento, lo que permite nuevos estudios^(8)^. Esta revisión del alcance se ha realizado de acuerdo con la lista de verificación *Preferred Reporting Items for Systematic Reviews and MetaAnalyses extension for Scoping Reviews* (PRISMA-ScR)^(9)^. El protocolo está registrado en el *Open Science Framework* con el identificador osf.io/j8bwa (https://doi.org/10.17605/OSF.IO/EM5TB).

### Pregunta Orientadora

Para elaborar la pregunta orientadora, se consideró el acrónimo PCC (Población, Concepto y Contexto), en el que Población (P) son los adolescentes y jóvenes; Concepto (C) es la adherencia a la terapia antirretroviral (TAR); y Contexto (C) es vivir con el VIH/sida. Así, se obtuvo la pregunta: ¿Cuáles son las evidencias científicas relacionadas con los determinantes sociales de la adherencia a la terapia antirretroviral entre adolescentes y jóvenes que viven con el VIH?

### Criterios de Inclusión

Se incluyeron estudios en los que la población estaba compuesta por adolescentes y jóvenes, considerando el rango de edad entre 10 y 24 años^(10)^. En cuanto al concepto, los estudios estaban relacionados con la adherencia al tratamiento antirretroviral, y la adherencia se midió explícitamente utilizando cualquier método, como por ejemplo la medición subjetiva (adherencia autorreferida), métodos fisiológicos (supresión viral) o mediciones farmacológicas (recuento de comprimidos, registros de reposición en farmacias), con el fin de encontrar el mayor número de estudios para identificar la laguna de conocimiento. En cuanto al contexto, fue amplio, ya que consideró la vida con el VIH en todos sus aspectos, tanto biológicos, psicológicos, sociales, culturales como espirituales, y sin restricción de localidad. Además, se consideraron los estudios publicados en su totalidad, sin restricción de idioma, límite temporal y diseño metodológico. También fueron elegibles los artículos publicados en revistas y en la literatura gris.

### Estrategia de Búsqueda

A partir de la búsqueda preliminar en el *Medical Literature and Retrieval System online* (MEDLINE) a través del *National Center for Biotechnology Information* (NCBI/PubMed) y *Web of Science,* se consideraron las palabras del texto contenidas en los títulos y resúmenes de los artículos relevantes para desarrollar la estrategia de búsqueda completa. A continuación, se realizó una búsqueda piloto final en dos bases de datos, a saber, MEDLINE a través de NCBI/PubMed y EMBASE. Tras estas etapas, se llevó a cabo la búsqueda definitiva y, para cada base de datos y/o fuente de información incluida, se adaptó la estrategia de búsqueda, incluidos los términos índice identificados y las palabras clave. Las búsquedas se realizaron en las bases de datos: *Web of Science* (WOS), MEDLINE a través de NCBI/PubMed, *Science Direct,* Scopus, EMBASE a través de *Elsevier*, *Scientific Electronic Library Online* (SciELO), COCHRANE y la Literatura Latinoamericana y del Caribe en Ciencias de la Salud (LILACS); Índice Bibliográfico Español en Ciencias de la Salud (IBECS); Base de Datos en Enfermería (BDENF); Index Medicus para el Pacífico Occidental (WPRIM); Literatura Peruana en Ciencias de la Salud (LIPECS); *World Health Organization’s Institutional Repository for Information Sharing* (WHO IRIS); Bibliografía Brasileña de Odontología (BBO) a través de la Biblioteca Virtual en Salud (BVS), a las que se accede a través del Portal de Revistas de la Coordinación de Perfeccionamiento del Personal de Nivel Superior (CAPES). Se consideraron como estrategias adicionales la búsqueda en fuentes de literatura gris: *Cybertesis*, *PeerJ Prepint*, *MedRxiv*, *OpenGrey*, *Preprints bioRxiv*, Catálogo de tesis y disertaciones, y la búsqueda de referencias cruzadas. Las búsquedas se realizaron entre julio y septiembre de 2024.

Para la construcción de la estrategia de búsqueda se utilizaron los operadores booleanos AND y OR, asociados a los descriptores MeSH (*Medical Subject Headings*), DeCS (Descriptores en Ciencias de la Salud), Emtree y descriptores no controlados (Cuadro 1).

**Cuadro 1:** Construcción, descriptores/palabras clave y operadores booleanos utilizados en la base de datos – Maringá, PR, Brasil, 2024.

Base de Datos	Estrategia de Búsqueda
*Web of Science*	ALL = ((((((“Adolescent”) OR (“Young Adult”)) AND ((“adherence to antiretroviral therapy”) OR (“Medication Adherence”) OR (“Cooperation and Adherence to Treatment”) OR (“Patient Cooperation”) OR (“antiretroviral therapy highly active”)) AND ((“HIV Infections”) OR (“HIV”) OR (“Acquired Immunodeficiency Syndrome”))))))
MEDLINE/PubMed	(((“Adolescent” OR “Young Adult”) AND (“adherence to antiretroviral therapy” OR “Medication Adherence” OR “Cooperation and Adherence to Treatment” OR “Patient Cooperation” OR “antiretroviral therapy highly active”) AND (“HIV Infections” OR “HIV” OR “Acquired Immunodeficiency Syndrome”)
Science Direct	(“Adolescent” OR “Young Adult”) AND (“adherence to antiretroviral therapy” OR “Medication Adherence” OR “Cooperation and Adherence to Treatment” OR “Patient Cooperation” OR “antiretroviral therapy highly active”) AND (“HIV” OR “Acquired Immunodeficiency Syndrome”)
Scopus	((“Adolescent”) OR (“Young Adult”) AND (“adherence to antiretroviral therapy”) OR (“Medication Adherence”) OR (“Cooperation and Adherence to Treatment”) OR (“Patient Cooperation”) OR (“antiretroviral therapy highly active”) AND (“HIV Infections”) OR (“HIV”) OR (“Acquired Immunodeficiency Syndrome”))
Embase	(“Adolescent” OR “Young Adult”) AND (“adherence to antiretroviral therapy” OR “Medication Adherence” OR “Cooperation and Adherence to Treatment” OR “Patient Cooperation” OR “antiretroviral therapy highly active”) AND (“HIV Infections” OR “HIV” OR “Acquired Immunodeficiency Syndrome”)
Scielo	((((((“Adolescent”) OR (“Young Adult”)) AND ((“adherence to antiretroviral therapy”) OR (“Medication Adherence”) OR (“Cooperation and Adherence to Treatment”) OR (“Patient Cooperation”) OR (“antiretroviral therapy highly active”)) AND ((“HIV Infections”) OR (“HIV”) OR (“Acquired Immunodeficiency Syndrome”))))))
COCHRANE	(“Adolescent” OR “Young Adult”) AND (“adherence to antiretroviral therapy” OR “Medication Adherence” OR “Cooperation and Adherence to Treatment” OR “Patient Cooperation” OR “antiretroviral therapy highly active”) AND (“HIV Infections” OR “HIV” OR “Acquired Immunodeficiency Syndrome”)
LILACS; IBECS; BDENF; WPRIM; LIPECS; WHO IRIS; BBO via BVS	(((((((“Adolescent”) OR (“Young Adult”)) AND ((“adherence to antiretroviral therapy”) OR (“Medication Adherence”) OR (“Cooperation and Adherence to Treatment”) OR (“Patient Cooperation”) OR (“antiretroviral therapy highly active”)) AND ((“HIV Infections”) OR (“HIV”) OR (“Acquired Immunodeficiency Syndrome”))))))) AND (db:(“LILACS” OR “IBECS” OR “BDENF” OR “WPRIM” OR “LIPECS” OR “WHOLIS” OR “BBO”))
*Cybertesis*	(“Adolescent” OR “Young Adult”) AND (“adherence to antiretroviral therapy” OR “Medication Adherence” OR “Cooperation and Adherence to Treatment” OR “Patient Cooperation” OR “antiretroviral therapy highly active”) AND (“HIV Infections” OR “HIV” OR “Acquired Immunodeficiency Syndrome”)
*PeerJ Prepint*	(“Adolescent” OR “Young Adult”) AND (“adherence to antiretroviral therapy” OR “Medication Adherence” OR “Cooperation and Adherence to Treatment” OR “Patient Cooperation” OR “antiretroviral therapy highly active”) AND (“HIV Infections” OR “HIV” OR “Acquired Immunodeficiency Syndrome”)
*MedRxiv*	(“Adolescent” OR “Young Adult”) AND (“adherence to antiretroviral therapy”) AND (“HIV” OR “Acquired Immunodeficiency Syndrome”)
*OpenGrey*	(“Adolescent” OR “Young Adult”) AND (“adherence to antiretroviral therapy” OR “Medication Adherence” OR “Cooperation and Adherence to Treatment” OR “Patient Cooperation” OR “antiretroviral therapy highly active”) AND (“HIV Infections” OR “HIV” OR “Acquired Immunodeficiency Syndrome”)
*Preprints bioRxiv*	(“Adolescent” OR “Young Adult”) AND (“adherence to antiretroviral therapy”) AND (“HIV” OR “Acquired Immunodeficiency Syndrome”)
Catálogo de tesis y disertaciones	(“Adolescent” OR “Young Adult”) AND (“adherence to antiretroviral therapy”) AND (“HIV” OR “Acquired Immunodeficiency Syndrome”)

### Selección de Fuentes

Tras las búsquedas en las fuentes de información, los resultados se exportaron a la aplicación *Rayyan*® del *Qatar Computing Research Institute* (QCRI). De este modo, de las 10 540 publicaciones, se excluyeron 1760 por duplicidad. A continuación, se sometieron a análisis 8779 estudios, que fueron seleccionados por dos revisores independientes en función de su título y resumen, con el fin de mantener el proceso de cegamiento; posteriormente, se compararon los estudios seleccionados y se llegó a un consenso. No fue necesaria la intervención de un tercer revisor. Tras ello, se excluyeron 8708 por no cumplir los criterios de inclusión.

Durante la lectura completa, se realizó una lectura en profundidad aplicando los criterios de elegibilidad. La información de los estudios se organizó y almacenó en hojas de cálculo electrónicas para garantizar una mejor interpretación y comparación entre los revisores.

Los artículos incluidos en la muestra final se organizaron en una herramienta de *Microsoft Excel*®, adaptada de acuerdo con la metodología JBI^(8)^, en la que se incluían los datos de caracterización de las publicaciones (autores, año, título, idioma, país, fuente de información, método, medida de adherencia, población, principales resultados).

### Análisis, Extracción y Presentación de los Datos

Se realizó una lectura minuciosa para clasificar los textos y, a continuación, se extrajeron los resultados. Además, se llevó a cabo una búsqueda manual para verificar la lista de referencias de los estudios incluidos en la revisión, lo que dio lugar a la inclusión de diez artículos más. El objetivo de esta etapa era encontrar estudios que no estuvieran vinculados a las bases de datos. Al final, formaron parte del presente estudio 39 publicaciones. La Figura 1 permite visualizar el proceso de búsqueda, exclusión y selección de las publicaciones según PRISMA^(9)^.

**Figura 1:** Flujograma de identificación y selección de artículos incluidos en la revisión del alcance. Maringá, PR, Brasil, 2024.

Fuente: adaptación de la lista de verificación PRISMA-ScR^(9)^.

Toda la información de los estudios incluidos se almacenó en hojas de cálculo y cuadros analíticos. Los estudios incluidos se analizaron a la luz de los Determinantes Sociales de la Salud^(11)^, lo que facilitó el proceso de interpretación y comparación de las producciones, ayudando así a la descripción de las evidencias y al mapeo de los datos, que se relacionaron con la descripción de los determinantes sociales de la adherencia entre adolescentes y jóvenes que viven con el VIH. Los determinantes se presentaron en forma de cuadro. Teniendo en cuenta que este estudio se trata de una revisión de alcance que utilizó publicaciones ya disponibles, no es necesaria la evaluación por parte de un Comité de Ética en Investigación. Sin embargo, la investigación se desarrolló teniendo en cuenta los aspectos éticos relacionados con la autoría de los artículos seleccionados.

## RESULTADOS

La aplicación de las estrategias de búsqueda en las fuentes de información permitió identificar 10540 publicaciones. Durante la selección de títulos y resúmenes, se obtuvieron 71 estudios para su análisis completo y, de estos, tras aplicar los criterios de elegibilidad, se seleccionaron 39 estudios cuyos resultados respondían a la pregunta principal ^(12–50)^.

### Características Generales de Los Estudios Incluidos

Se observó que 37 (94,87%) ^(12–21,23–50)^ presentaban el texto disponible en inglés, uno (2,56%) en francés^(22)^ y uno (2,56%) en portugués^(18)^. El período de publicación abarcó los años 1999 a 2024, con 20 publicaciones relativas al período 2017–2024 ^(31–50)^. De los 39 estudios, 17 informaron haber sido desarrollados en el continente africano: cinco en Uganda ^(24,30,39,45–46)^, tres en Sudáfrica ^(42,44,50)^, dos en Botsuana ^(21,37)^, Kenia ^(36,40)^, Etiopía ^(47, 48)^ y Zambia ^(35, 36)^, uno en cada uno de los siguientes países: Zimbabue^(27)^, África Subsahariana^(28)^, Malaui^(32)^, Camerún^(41)^, Nigeria^(43)^ y Esuatini^(49)^; en América del Norte se han desarrollado 15 estudios, todos ellos en los Estados Unidos de América ^(12–17,19–20,23,25,26,29,31,33,38)^; en América Latina solo se ha elaborado un estudio en Brasil^(18)^; y en Europa se ha encontrado una publicación procedente de Bélgica^(22)^.

### Objetivos, Métodos, Medidas de Adherencia y Participantes de los Estudios Incluidos.

En cuanto a los objetivos de los estudios, la mayoría de las publicaciones se diseñaron para determinar los factores asociados a la adherencia al tratamiento del VIH entre adolescentes y jóvenes, en relación con los resultados promotores y limitantes de la continuidad del seguimiento. Otras publicaciones se centraron en la evaluación de la adherencia, así como en las experiencias de los pacientes en relación con las barreras y los facilitadores de la adherencia.

En cuanto a los métodos utilizados en los estudios, se observó que 28 (71,79%) son cuantitativos ^(12–17,19–23,26–30,32,34,38–41,44,46,47)^, siendo 23 con diseño transversal ^(12–16,20–23,26–30,32,34,38–42,44,46,47,49,50)^, tres prospectivos ^(17,19,50)^, uno retrospectivo^(42)^ y uno longitudinal retrospectivo^(49)^; cinco (12,82%) cualitativos ^(18,24,31,37,45)^, siendo uno de tipo fenomenológico^(45)^ y cuatro descriptivos ^(18,24,31,37)^; tres (7,69%) métodos mixtos ^(35,36,43)^, de los cuales solo uno se clasificó como paralelo convergente^(24)^, dos (5,12%) revisiones sistemáticas con metaanálisis ^(25,48)^ y uno (2,58%) de enfoque cuantitativo y cualitativo^(33)^. En cuanto a las medidas de adherencia, se mencionaron 51 formas, siendo 35 (68,62%) de medicación subjetiva orientada a la adherencia autorreferida ^(12,14–17,19–46,48,50)^, nueve (17,64%) de métodos fisiológicos ^(14,18,22,23,25,33,42,49,50)^ y siete (13,72%) de mediciones farmacológicas ^(13,21,24,25,45,48,50)^. De los 39 estudios, se observó que 28 (71,80%) utilizaron solo una medida de adherencia ^(12–17,19–21,26,27,29–32,34–40,43–47,50)^, ocho (20,51%) utilizaron dos medicamentos ^(18,22–24,41,42,48,49)^ y tres (7,69%) estudios utilizaron tres medidas de adherencia ^(25,28,33)^. En cuanto a los participantes, solo se realizó un estudio con adolescentes y jóvenes junto con sus familiares^(33)^. En cuanto al rango de edad de los participantes, se observó que 15 (38,47%) estudios incluían adolescentes y jóvenes (10–24 años) ^(12,13,16,18–20,22,23,26,29,31,38,43,45,47)^ y 24 (61,53%) solo adolescentes (10–19 años) ^(14,15,17,21,24,25,27,28,30–37,39–42,44,46,48,49,50)^.

### Estructura Conceptual

Los factores promotores y limitantes identificados en los estudios incluidos se organizaron en cinco dimensiones relacionadas con los determinantes que influyen en la adherencia al tratamiento entre adolescentes y jóvenes que viven con el VIH: Dimensión 1: Características individuales; Dimensión 2: Estilo de vida y comportamientos individuales; Dimensión 3: Redes sociales y comunitarias; Dimensión 4: Condiciones de vida y de salud; Dimensión 5: Condiciones socioeconómicas, culturales y ambientales. Los determinantes se resumen en la Cuadro 2.

**Cuadro 2:** Resumen de los determinantes identificados en los estudios incluidos (n = 39) en la revisión del alcance. Maringá, PR, Brasil, 2024.

Dimensión de los determinantes	Determinantes sociales de la adherencia al tratamiento antirretroviral entre adolescentes y jóvenes que viven con el VIH	
	Promotores	Limitantes
Dimensión 1: Características individuales	**1. Comprender la salud y el tratamiento:** Aumento de los conocimientos sanitarios^(23)^ Conocimiento del estado serológico ^(25,48)^ Comprender la enfermedad^(26)^ Comprender la importancia de la medicación^(31)^ Conocimiento de la eficacia del TAR^(36)^ Percepción de la utilidad de los medicamentos contra el VIH^(45)^ **2. Motivación y autoeficacia:** Esperan prolongar su vida útil al menos 10 años^(12)^ Motivadores personales orientados a objetivos^(31)^ Deseo de estar sano y vivir^(37)^ Disposición motivacional para el tratamiento antirretroviral^(38)^ Mayor autoeficacia ^(38, 40)^ Formas de automotivarse para lograr la adherencia^(43)^ **3. Estrategias de gestión del tratamiento:** Uso de ayudas para la memoria^(31)^ Uso de recordatorios^(43)^ Estrategias de ocultación^(43)^ Saben instintivamente cuándo es el momento de utilizar sus antirretrovirales^(43)^ **4. Actitudes y creencias personales:** Miedo a las consecuencias negativas de la no adherencia^(34)^ Satisfacción con la vida^(46)^	**1. Factores relacionados con la edad y el desarrollo:** Jóvenes mayores ^(17,33,42,46)^ Retraso del crecimiento^(25)^ **2. Olvido y falta de coherencia:** Olvido ^(20,22,32–34,37)^ El adolescente nunca toma TAR mientras está fuera^(25)^ Haber omitido dosis de TAR por no recogerlo en la farmacia^(21)^ **3. Falta de motivación y rechazo del tratamiento antirretroviral:** No tener ganas de tomar la medicación^(20)^ No quieren que se les recuerde que están infectados por el VIH^(20)^ Baja autoeficacia en el tratamiento^(32)^ A los adolescentes no les importan las TAR ^(25)^ **4. Factores contextuales:** Hombre ^(21, 25, 46)^ Mujer^(47)^ Ser huérfano por partida doble^(25)^ Falta de conocimientos básicos sobre el VIH^(35)^ Adolescentes con VIH adquirido horizontalmente^(42)^
Dimensión 2: Estilo de vida y comportamiento individual	**1. Estabilidad y rutina de vida:** Estabilidad de las situaciones de vida^(13)^ Estilo de vida y rutina diaria^(31)^ Adolescentes en trayectorias de adherencia con inicio bajo y creciente^(50)^ **2. Salud mental y bienestar:** Bienestar y bajo malestar psicológico ^(29,38,44)^ Menor consumo de alcohol ^(17,29)^	**1. Problemas de salud mental:** Depresión ^(14,16,17,19,24,35)^ Sufrimiento psicológico ^(20,29)^ Puntuación baja en salud mental^(25)^ Unidad de aumento del malestar psicológico^(28)^ Problemas emocionales^(31)^ Sentirse deprimido/sobrecargado^(32)^ Infelicidad autodeclarada^(33)^ **2. Consumo de sustancias:** Consumo de sustancias ^(12,20,38)^ Consumo de marihuana ^(16,29)^ Consumo de alcohol^(32)^ **3. Comportamientos y actividades de distracción:** Estar ocupado con otras cosas^(32)^ Tener novio/a, chatear en línea con frecuencia^(33)^ **4. Comportamiento de incumplimiento:** Negativa intencionada a tomar la medicación^(43)^ Ser sexualmente activo^(25)^
Dimensión 3: Redes sociales y comunitarias	**1. Apoyo familiar:** Apoyo familiar^(18)^ Administración de medicamentos por los padres^(25)^ Cohesión familiar^(39)^ Comunicación adolescente-cuidador^(39)^ Revelaron su estado serológico respecto al VIH (madre)^(12)^ Tener un cuidador con pareja^(25)^ Que el responsable esté presente en cada reunión clínica^(27)^ **2. Apoyo de los profesionales sanitarios:** Apoyo del equipo sanitario^(18)^ Profesionales sanitarios que prestan apoyo^(24)^ Adolescentes a los que un profesional sanitario enseñó a tomar el TAR^(25)^ Comodidad para hacer preguntas al profesional sanitario^(27)^ **3. Apoyo social entre compañeros y grupos:** Apoyo entre iguales^(24)^ Asesoramiento de profesionales sanitarios^(24)^ Apoyo social y emocional y asesoramiento de grupos de iguales^(33)^ Apoyo de los compañeros de tratamiento o de la familia^(43)^ Servicios de apoyo y asesoramiento social^(36)^ Recursos comunitarios para el VIH^(36)^ Mayor apoyo social ^(38,45)^ **4. Estrategias de afrontamiento y recursos:** *Afrontamiento* (estrategias de *afrontamiento* proactivas; recurrir a la familia; *afrontamiento* espiritual; ayuda profesional)^(19)^ Apoyo en el entorno familiar^(37)^ No tomar la medicación en la escuela^(17)^ El adolescente no conoce los problemas de salud del cuidador^(25)^	**1. Características del cuidador:** Cuidador viudo^(25)^ El cuidador no tiene una práctica religiosa^(25)^ Escasa participación de los cuidadores^(25)^ Bajo nivel educativo del cuidador^(25)^ Baja capacidad intelectual evaluada por el cuidador^(33)^ Tener abuelos o parientes como cuidadores principales^(33)^ Tener un abuelo como cuidador principal^(46)^ El cuidador es el único que conoce el estado serológico del niño^(25)^ **2. Deterioro de la dinámica familiar y relacional:** Tener una familia desorganizada y acuerdos in loco *parentis* ^(25)^ Relaciones tensas con los cuidadores^(33)^ Falta de atención, apoyo y amor por parte del padre^(43)^ Culpar a la persona que contaminó a la madre^(43)^ **3. Violencia y trauma:** Presenciar o sufrir violencia en el hogar^(32)^ Pérdida de la madre^(35)^ **4. Comunicación y colaboración:** Mala comunicación con el médico^(33)^ Falta de colaboración entre organizaciones y normas sociales^(36)^
Dimensión 4: Condiciones de vida y de salud	**1. Aspectos clínicos del tratamiento:** Nivel de CD4 ≥ 500 células/mm3^(13)^ Aumento del recuento de CD4+^(34)^ Carga viral <1000 copias/ml^(40)^ Carga viral basal indetectable^(49)^ Tomar profilaxis con cotrimoxazol junto con TAR^(25)^ Número de medicamentos prescritos^(13)^ Falta de efectos secundarios y nutrición/dietas^(31)^ Intimidad para tomar el medicamento^(31)^ Características de los medicamentos (número y tamaño de los comprimidos, régimen)^(31)^ Una vez al día, la dosis^(40)^ **2. Recursos para las condiciones de vida y la salud:** Participar en sesiones de grupo dirigidas por un animador profesional^(27)^ Suministro de alimentos^(24)^ Comida suficiente mientras se toma la medicación^(44)^ Suministro adecuado de medicamentos^(36)^ Aquellos a los que se atendió en la unidad^(47)^ **3. Accesibilidad a los servicios sanitarios:** Poco tiempo de espera^(24)^ Vivir más cerca de una clínica^(30)^ Disponibilidad/accesibilidad (disponer de transporte) ^(24,31)^ Dinero suficiente para viajar a la clínica^(44)^ Viajar cerca de casa^(32)^ Disponibilidad de servicios gratuitos^(45)^	**1. Malas condiciones de vida y alimentación:** Inestabilidad o falta de vivienda ^(12,13)^ Falta de nutrición adecuada^(36)^ **2. Aspectos relacionados con el tratamiento:** Muchas pastillas ^(12,24)^ Tomar 3 o más comprimidos al día^(25)^ Dificultad del cuidador para administrar la medicación^(25)^ Medicación administrada por el adolescente^(25)^ Adolescentes en la fase más avanzada del VIH, que presentan SIDA^(17)^ Antecedentes de infección oportunista^(47)^ Carga viral de ARN ≥1000 copias/ml^(33)^ Carga viral basal detectable^(49)^ No seguir el régimen prescrito^(28)^ **3. Efectos secundarios y carga del tratamiento:** Efectos adversos relacionados con la medicación ^(15,24,35,41,43)^ Intolerancia a los medicamentos^(25)^ Fatiga medicamentosa ^(24,31)^ Cansado de tomar medicación todos los días durante el resto de su vida^(43)^ Carga percibida del tratamiento^(45)^ Efecto percibido de la terapia antirretroviral en el aspecto físico^(43)^ **4. Acceso y apoyo deficientes por parte de los servicios sanitarios:** Vivir lejos del trabajo, ya sea en zonas urbanas o rurales^(25)^ Vivir en zonas remotas^(50)^ Baja satisfacción con la asistencia sanitaria^(19)^ Falta de servicios específicos para adolescentes^(36)^ El cuidador no paga por el tratamiento^(25)^ Complicaciones en las rutinas diarias^(15)^ Falta a una cita médica en los últimos 6 meses^(32)^ Ha omitido una o más dosis de TAR en la última semana^(36)^ Largos tiempos de espera en la clínica^(34)^
Dimensión 5: Condiciones socioeconómicas, culturales y medioambientales	**1. Recursos económicos:** Mayor propiedad de activos, concretamente propiedad familiar de siete o más activos materiales^(30)^ Mayor ventaja económica en la posesión de enseres domésticos, ahorros financieros y empleo como cuidador^(30)^ Mejoras en el bienestar económico dentro de las personas y entre ellas^(44)^ **2. Reducción del estigma:** Reducir el estigma interiorizado del VIH^(44)^	**1. Estigma y discriminación:** Prejuicios, discriminación y estigmatización ^(18,24,34,36,37)^ Estigma interiorizado^(41)^ Sentirse estigmatizado por la gente de fuera y de dentro de casa^(32)^ Estigma y discriminación percibidos^(45)^ **2. Temores relacionados con la revelación:** Miedo a ser visto tomando el antirretroviral ^(22,24)^ Miedo a la revelación ^(31,36,37,43)^ Miedo a revelar el estado serológico a otras personas, especialmente a los chicos/novias^(33)^ **3. Condiciones socioeconómicas:** Pobreza ^(24,36)^

Fuente: Datos de la investigación, 2024.

Esta revisión identificó que los factores relacionados con las características individuales, el estilo de vida, las redes sociales y comunitarias, las condiciones de vida y de trabajo, las condiciones socioeconómicas, culturales y ambientales de los adolescentes y jóvenes influyeron en la adherencia al tratamiento del VIH, y que estos factores se ven constantemente moldeados por ser multinivel, es decir, interactúan de forma compleja. Así, los determinantes sociales de la adherencia afectan al contexto del tratamiento antirretroviral, tanto de forma positiva como negativa, y están directamente relacionados con las condiciones de vida de los adolescentes y jóvenes que viven con el VIH (Figura 2).

**Figura 2:** Síntesis conceptual de los resultados obtenidos de la muestra final de los determinantes sociales de la adherencia entre adolescentes y jóvenes que viven con el VIH. Maringá, Paraná, Brasil, 2024.

Fuente: Datos de la investigación, 2024.

## DISCUSIÓN

Los estudios incluidos en la revisión abarcan una amplia gama de países y continentes, como América del Norte, América Latina, África, Asia y Europa, con países de ingresos medios y medios-altos per cápita. Las diversidades geográficas y culturales son importantes para comprender cómo los diferentes contextos sociales, económicos y culturales influyen en la adherencia al tratamiento antirretroviral entre adolescentes y jóvenes. La adaptación de las estrategias de intervención debe tener en cuenta estas variaciones contextuales^(51)^.

En cuanto a la temporalidad de los materiales encontrados, una parte significativa de los estudios se publicó entre los años 2017 y 2024. Esto se debe especialmente al lanzamiento de la Declaración Política de la Asamblea General de las Naciones Unidas sobre el fin del Sida y la reducción de las nuevas infecciones por el VIH. A partir de la publicación de esta declaración, en 2016, los países de todo el mundo se comprometieron a acelerar sus acciones de lucha contra el VIH y el sida para acabar con la epidemia para el año 2030^(52)^.

Se observa que la mayoría de los estudios no han adoptado un enfoque cualitativo, que es esencial para explorar las barreras y los facilitadores de la adherencia al TAR entre los adolescentes y los jóvenes. El enfoque cualitativo permite un análisis más profundo de las percepciones, experiencias y contextos sociales de los adolescentes y jóvenes que viven con el VIH, además de esclarecer cuestiones psicosociales y culturales que afectan a la adherencia, lo que puede no ser captado por estudios cuantitativos ^(53,54)^.

En relación con las características individuales de los adolescentes y jóvenes que viven con el VIH, se observó que un mayor conocimiento del estado serológico y de la importancia de los antirretrovirales son factores determinantes que promueven la adherencia. De este modo, si el joven tiene acceso a la información y la comprende, esto le lleva a tener conocimientos sobre el VIH/sida y la TARV. Estas evidencias ayudan a los gestores a tomar decisiones sobre la formación y la actualización de los consejeros y profesionales de la salud para que, a su vez, puedan transmitir información precisa a los adolescentes y jóvenes^(55)^.

Así, la educación en salud ha sido un factor que influye en la adherencia al tratamiento de numerosas infecciones y enfermedades, en especial el VIH y el sida. Los pacientes que reciben información adecuada a su nivel de comprensión obtienen resultados significativos a lo largo del tratamiento ^(56,57)^. El conocimiento y las creencias sobre el VIH de los adolescentes y jóvenes que viven con el VIH desempeñan un papel esencial en su experiencia^(58)^.

Junto con estos factores individuales, los objetivos personales y el deseo de vivir, sumados a la satisfacción con la vida, son aspectos que promueven la adherencia al tratamiento. Un estudio transversal realizado en China señaló que las personas que aceptan el diagnóstico de VIH se adhieren al tratamiento y lo siguen correctamente. En consecuencia, tienen una mayor calidad y esperanza de vida^(59)^. La aceptación del VIH como una enfermedad crónica ayuda a cambiar el comportamiento de los adolescentes y los jóvenes y, a su vez, los lleva a un cambio de estilo de vida, con el objetivo de volverse indetectables e intransmisibles (I=I)^(58)^.

Sin embargo, algunas características personales dificultan la realización de un tratamiento correcto, como la edad avanzada y el sexo masculino. Así, ser joven ya es un factor, ya que su responsabilidad se considera una barrera, relacionada con el cansancio de realizar un tratamiento continuo, además de la comparación con personas del mismo círculo social y la participación en otras actividades y costumbres propias de la edad. Además, los jóvenes varones tienen más dificultades para buscar ayuda en los servicios de salud, debido al propio estereotipo de género, lo que perjudica la participación de los hombres en toda la cascada de atención. De este modo, al tratarse de una etapa en la que reciben menos apoyo social y familiar, los adolescentes mayores y los jóvenes son más propensos a no adherirse al tratamiento o a tener una mala adherencia^(57)^.

Además, una revisión sistemática y un metaanálisis realizados con adolescentes huérfanos mostraron que la condición de huérfano es una barrera significativa para la adherencia al TARV, especialmente en países de bajos ingresos. La dependencia de cuidadores, que puede ser inconsistente o insuficiente, agrava este desafío, lo que indica la necesidad de intervenciones que aumenten el apoyo social y mejoren la educación de los cuidadores sobre la importancia de la adherencia al tratamiento^(60)^.

A esto se suma que muchos adolescentes y jóvenes presentan lapsos ocasionales en la toma de sus medicamentos antirretrovirales debido al olvido, que a menudo es causado por el estrés psicológico de tener la infección y/o la enfermedad y tener que tomar el medicamento a diario^(61)^.

En cuanto a la dimensión del estilo de vida y los comportamientos individuales, se ha observado que el bienestar psicológico es un determinante importante que debe tenerse en cuenta en las personas que viven con el VIH, especialmente en los adolescentes y los jóvenes, ya que es uno de los factores que contribuyen al éxito del tratamiento. Estas personas se dedican al tratamiento con el fin de prolongar su vida y adoptan un estilo de vida saludable, con una rutina que minimiza el sufrimiento psicológico y les permite experimentar situaciones de resiliencia frente a su condición^(62)^.

Sin embargo, los trastornos mentales y el sufrimiento psicológico, como la ansiedad y la depresión, son habituales en la vida de estos jóvenes, lo que, sumado al abuso de sustancias lícitas e ilícitas, puede llevar al abandono del tratamiento^(62)^. De este modo, experimentar disonancia emocional y tener dificultades para aceptar la propia identidad muestran que la salud mental es frágil, lo que repercute en el tratamiento^(58)^.

En la dimensión de las redes sociales y comunitarias, se observó que el apoyo, ya sea social, familiar o del profesional de la salud, es un factor determinante que contribuye positivamente a la adherencia a la terapia antirretroviral entre los adolescentes y jóvenes que viven con el VIH. Las relaciones sociales son necesarias para todos los individuos, especialmente para las personas que viven con una enfermedad crónica, en la que la red de apoyo y las relaciones de confianza son importantes para afrontar la enfermedad^(63)^. Un estudio realizado con adolescentes peruanos destacó cómo los factores sociales y culturales, incluido el apoyo familiar y las dinámicas sociales, influyen en la adherencia al tratamiento. El uso de modelos ecológicos sociales en estos estudios enfatiza la necesidad de considerar múltiples niveles de influencia, desde el nivel individual hasta el comunitario, al diseñar intervenciones para mejorar la adherencia al TAR^(53)^.

Además, las relaciones de confianza establecidas con los profesionales de la salud son importantes para algunos pacientes, siendo, en la mayoría de los casos, la única fuente de apoyo y orientación^(57)^. Una revisión sistemática de la literatura identificó que el asesoramiento y la educación sobre la terapia antirretroviral son facilitadores de la adherencia al tratamiento, al considerar al paciente en su totalidad, incluyendo los aspectos étnicos, culturales, socioeconómicos y el nivel educativo del individuo ^(56,57)^.

En este mismo sentido, el apoyo entre pares es importante para afrontar el tratamiento; sin embargo, algunos jóvenes se sienten avergonzados y diferentes cuando se relacionan con compañeros que no tienen la misma condición, por lo que no encuentran apoyo mutuo^(64)^. De este modo, algunos adolescentes y jóvenes que viven con el VIH buscan continuamente y pueden haber recibido apoyo de otras personas significativas y de la comunidad en su vida, lo que hace que el tratamiento y el diagnóstico se conviertan en algo más llevadero en su día a día^(58)^.

La dinámica familiar deteriorada y la relación de un joven que vive con el VIH con los demás miembros de la familia y los traumas en el entorno doméstico siguen siendo factores determinantes que han influido negativamente en la adherencia al tratamiento antirretroviral. El estigma y los prejuicios, en la mayoría de los casos, provienen de la propia familia y corroboran los síntomas depresivos y las ideas suicidas^(57)^.

Por lo tanto, es imprescindible invertir en apoyo social para estos pacientes, junto con sus familiares que conocen el diagnóstico y el equipo de salud, a fin de mejorar su calidad de vida y supervivencia a partir de la adherencia al TAR^(65)^. Además, cabe destacar que solo un estudio^(33)^ aportó la perspectiva del cuidador familiar. Este resultado remite a la necesidad de desarrollar nuevos estudios en este ámbito con el fin de mejorar la adherencia a la terapia antirretroviral junto con el apoyo de los familiares de adolescentes y jóvenes que viven con el VIH.

Se observa que, en la dimensión de condiciones de vida y salud, los determinantes promotores están relacionados con el tratamiento y la accesibilidad a los servicios de salud. Se puede identificar que una carga viral <1000 copias/ml o indetectable y un recuento de CD4+ ≥ 500 células/mm3 o aumentado se asocia con la adherencia, ya que los jóvenes se sienten motivados a continuar el tratamiento. La supresión del VIH es una estrategia esencial para optimizar la salud y el bienestar de las personas que viven con la infección. Las pruebas rutinarias de carga viral (CV) y recuento de CD4+ son esenciales para mejorar las oportunidades de tratamiento y son una herramienta particularmente útil entre los grupos con alto riesgo de fracaso virológico, como los adolescentes^(66)^.

Un estudio realizado en el Reino Unido e Irlanda mostró que en los jóvenes que iniciaron el TAR antes de los 10 años, el recuento de CD4+ disminuyó a partir de los 10 años y que la supresión viral <400 copias/ml a lo largo del tiempo presentó recuentos medios de CD4+ que se acercaban a la inmunodeficiencia leve (350–500 células/mm3) a los 20 años^(67)^.

Los efectos adversos de los antirretrovirales se consideraron un factor limitante de la adherencia al TARV, ya que los adolescentes y jóvenes que viven con el VIH a menudo luchan con el autocontrol del tratamiento. Muchos presentan síntomas debidos a la enfermedad del VIH, afecciones comórbidas y/o efectos adversos de los antirretrovirales^(62)^.

Los efectos adversos influyen directamente en la adherencia, ya que afectan a la vida cotidiana del adolescente y/o joven y, en consecuencia, a su calidad de vida. Por lo tanto, para neutralizar las barreras y, al mismo tiempo, mejorar los facilitadores, la optimización del tratamiento con regímenes basados en dolutegravir (DTG), los enfoques orientados por pares, como los compañeros de apoyo para jóvenes y adolescentes, y los modelos diferenciados de prestación de servicios, incluida la dispensación de varios meses, es una excelente estrategia para minimizar el impacto del tratamiento en sus vidas ^(68–70)^.

La distancia geográfica ha sido un factor preponderante en la adherencia al tratamiento y la atención del VIH, principalmente debido a su cronicidad. En su mayoría, es un factor que limita el acceso a los servicios de salud, siendo una barrera que interfiere directamente en las tasas de adherencia y abandono del tratamiento cuando las distancias son mayores ^(65,71)^. La falta de recursos económicos también se refleja en el acceso al tratamiento y, sumada a la distancia, puede interferir tanto en los resultados de la propia salud del individuo como en la dinámica de los servicios, ya que estos acaban faltando a las consultas y rutinas o abandonando el tratamiento ^(65,72)^.

Del mismo modo, la accesibilidad a los servicios de salud asociada a la distancia del lugar de residencia y a los factores socioeconómicos de las personas son barreras para una buena adherencia al TARV, lo que aumenta la vulnerabilidad de los adolescentes^(57)^. Por el contrario, cuando los adolescentes y jóvenes viven cerca del servicio de VIH, acuden con más frecuencia, precisamente porque no tienen que pagar por la atención y porque residen cerca del lugar de consulta y de recogida de la medicación ^(30,45)^.

Por lo tanto, es importante que el servicio especializado en VIH esté integrado a los demás niveles de servicios de salud, en especial a los que componen la atención primaria, ya que la falta de integración ha dificultado el desarrollo de estrategias y acciones de prevención y promoción para esta población^(71)^.

Dicho esto, es esencial que los servicios de salud dedicados al VIH también realicen un seguimiento de las ausencias y sus motivos, y así busquen alternativas para una mayor adherencia a las consultas de seguimiento y al uso de la TARV. Además, se debe concienciar a los pacientes para que se responsabilicen de su propia salud, de modo que se impliquen en su cuidado y, en consecuencia, mejoren su calidad de vida^(73)^.

Por otra parte, las condiciones socioeconómicas, culturales y ambientales ponen de manifiesto la necesidad de que la sociedad y los adolescentes y jóvenes que viven con el VIH reduzcan los estigmas. Un estudio realizado en Filipinas mostró que lidiar con los estereotipos sobre el VIH contribuyó a la forma en que los adolescentes y jóvenes asumen los juicios de los demás (estigma percibido) y, así, internalizan el estigma que altera su creencia sobre sí mismos y sobre la enfermedad^(58)^. También se observa que el estigma y la discriminación son causados por la desinformación sobre la enfermedad y provienen tanto de ellos mismos como de otras personas de su entorno^(58)^.

Hay pruebas de que los prejuicios, la discriminación, el estigma y el miedo a revelar el diagnóstico han perjudicado significativamente la adherencia al tratamiento antirretroviral. Un estudio realizado con adolescentes en Etiopía destacó que las barreras del estigma, el prejuicio, la discriminación y el miedo a revelar el diagnóstico han sido las más prevalentes. Por lo tanto, la investigación insta a intervenciones dirigidas a mejorar el apoyo social y reducir el estigma, el prejuicio y la discriminación, de modo que los adolescentes y los jóvenes dejen de lado el miedo y puedan convivir mejor con el VIH^(74)^.

Estudios cualitativos recientes, como los realizados en países del África subsahariana y en Perú ^(53,54)^, destacan el papel fundamental del estigma, el miedo a la divulgación y los retos emocionales a los que se enfrentan los adolescentes, que son determinantes para la adherencia óptima al TAR. Cabe destacar que la pandemia de COVID-19 ha exacerbado estas barreras, aumentando el aislamiento social y dificultando el acceso a los servicios de salud, lo que ha tenido un impacto negativo en la adherencia al TAR en varias regiones del mundo^(75)^.

La pobreza también ha sido un fenómeno profundamente estigmatizado debido a los aspectos de desigualdad social, además de ser un factor que contribuye a la inseguridad alimentaria y de vivienda, lo que repercute negativamente en la adherencia al tratamiento. Por lo tanto, es esencial abordar la inseguridad de los recursos para poder llegar al núcleo del estigma relacionado con el VIH^(76)^.

Los adolescentes y jóvenes que viven con el VIH en países de ingresos bajos y medios se encuentran en contextos geográficos y socioeconómicos diferentes; en consecuencia, corren un mayor riesgo de sufrir problemas psicosociales, de desarrollo y comorbilidades, lo que los convierte en un grupo especialmente vulnerable a la adherencia al tratamiento. Además, también existen discrepancias entre los escenarios de recursos, la falta de estructura y la comunicación ineficaz entre los proveedores de atención sanitaria^(77)^.

Todas estas dimensiones tienen implicaciones para las estrategias de promoción de la salud pública en el contexto de la adherencia al tratamiento de los adolescentes y jóvenes que viven con el VIH, incluyendo el diseño y el desarrollo de intervenciones basadas en el hogar, la sociedad y los profesionales de la salud. Las estrategias eficaces deben ser multifacéticas y abordar los factores conductuales, sociales y estructurales para minimizar los casos de mala adherencia y pérdida de seguimiento.

En este contexto se inscriben los esfuerzos internacionales, con el objetivo de acabar con el VIH como problema de salud pública para 2030, con la propuesta de combatir las desigualdades sociales y ampliar las acciones intersectoriales. Alcanzar la meta 95-95-95 debe ser el objetivo de todos los países del mundo, y para ello es necesario que se disponga de pruebas y tratamiento oportunos, además de la remisión de la carga viral. Además de la adherencia al tratamiento farmacológico, también es necesario que los adolescentes y jóvenes que viven con el VIH adopten una actitud de autocuidado activo, mediante la adopción de un estilo de vida saludable, acudiendo a las consultas, realizándose las pruebas, retirando los medicamentos en la fecha prevista y tomándolos diariamente de forma correcta. De este modo, el centro de la atención son los adolescentes y jóvenes que viven con el VIH; por lo tanto, los profesionales de la salud deben fomentar una actitud activa en el propio tratamiento^(78)^.

### Limitaciones del Estudio

Como limitación, cabe destacar el hecho de que se han tenido en cuenta todo tipo de medidas de adherencia, lo que implica posibles sesgos en la muestra, así como la heterogeneidad metodológica asociada a cada tipo de medida, lo que imposibilita la generalización de los datos. Otra limitación es la no inclusión de textos en otras bases de indexación; de este modo, se reconoce que pueden haberse omitido importantes investigaciones publicadas con la estrategia de búsqueda utilizada en esta revisión. Sin embargo, cabe destacar que se han seguido todos los rigores científicos exigidos para una revisión de alcance de acuerdo con las recomendaciones del JBI. Además, los resultados de la síntesis conceptual deben interpretarse con cautela, ya que presentan diferentes escenarios epidemiológicos y socioeconómicos, así como medidas y estrategias implementadas, aspectos operativos y organizativos, y el formato del sistema de salud en cada país.

### Avances Para el Área de Enfermería y Salud

Los profesionales de la salud, en especial los enfermeros que trabajan en ambulatorios de VIH y en la Atención Primaria de Salud (APS) atendiendo a adolescentes y jóvenes, pueden beneficiarse de los resultados, ya que el marco conceptual sintetizado pone de relieve situaciones cotidianas que influyen directamente en el tratamiento. Por lo tanto, una vez reconocidos los factores determinantes que promueven y limitan la adherencia al tratamiento, la búsqueda de estrategias hace que la reducción de las tasas de mala adherencia y no adherencia al tratamiento sea menos ardua y más específica. A partir de este estudio, se pueden realizar nuevas investigaciones con el fin de eliminar los factores que se sabe que limitan el tratamiento de los adolescentes y jóvenes que viven con el VIH en varios frentes y en diferentes contextos.

## CONCLUSIÓN

El mapeo de los determinantes que promueven y/o limitan la adherencia a la terapia antirretroviral entre adolescentes y jóvenes que viven con el VIH ha permitido mitigar la (re)estructuración de las políticas públicas en relación con la práctica clínica, especialmente en los servicios de salud que atienden las necesidades de este público, en la reducción de las desigualdades sociales y en la promoción de la adherencia al tratamiento, lo que, en consecuencia, conduce al cumplimiento de los objetivos para 2030. Los hallazgos sintetizados destacan que la adherencia al tratamiento se basa en las creencias y conocimientos personales, en la dinámica de la rutina en el entorno familiar y en el acceso a los servicios de salud. Este estudio también plantea la necesidad de realizar nuevas investigaciones que busquen captar la diversidad de factores que se reflejan en la experiencia vivida y los comportamientos relacionados con la adherencia al tratamiento antirretroviral en el contexto de la adolescencia y la juventud.

## DISPONIBILIDAD DE DATOS

El conjunto de datos que respalda los hallazgos de este estudio no está disponible públicamente, sin embargo, todos los datos que respaldan los hallazgos de este estudio están disponibles a pedido del autor correspondiente.
